# Prevalence of anxiety and depression in breast cancer patients: a meta-analysis

**DOI:** 10.3389/fpsyg.2026.1869669

**Published:** 2026-10-14

**Authors:** Chao-Yi Jia, Zhao-Yue Chen, Bei-Te Liu, Jing-Lu Bai, Qing-Ze Chen, Hao-Wen Zhang, Jia-Pu Bi, Jin-Peng Zhang, Wen-Qiong Liu, Meng-Zhen Tian

**Affiliations:** 1 Department of Traditional Chinese College, Shandong University of Traditional Chinese Medicine, Jinan, Shandong, China; 2 Department of Acupuncture and Massage College, Shandong University of Traditional Chinese Medicine, Jinan, Shandong, China; 3 Department of Pharmacy College, Shandong University of Traditional Chinese Medicine, Jinan, Shandong, China; 4 Shandong Provincial Hospital of Traditional Chinese Medicine, Jinan, Shandong, China

**Keywords:** anxiety, breast cancer, depression, meta-analysis, prevalence

## Abstract

**Introduction:**

Depression and anxiety are frequently reported among women with breast cancer, but prevalence estimates vary substantially across settings and assessment instruments. This meta-analysis synthesized recent international evidence and examined overall, severity-stratified, and instrument-specific prevalence estimates.

**Methods:**

We searched PubMed, Web of Science, Embase, the Cochrane Library, APA PsycINFO, and Scopus for observational studies published from January 2015 through September 10, 2026. Proportions were pooled with a random-effects model after Freeman-Tukey double-arcsine transformation. Prespecified subgroup analyses examined region, assessment instrument, and study design; publication-year meta-regression and formal tests for small-study effects were also conducted. A quality-threshold sensitivity analysis re-included all otherwise eligible quality-appraised reports with extractable event counts and denominators.

**Results:**

Sixty-nine reports underwent quality appraisal; 52 entered the primary meta-analysis and 17 did not exceed the prespecified quality thresholds. The 52 reports represented 16,850 independent participants. Depression data comprised 49 reports and 16,504 participants, and anxiety data comprised 39 reports and 11,536 participants. The pooled prevalence was 44.3% for depression (95% CI: 38.3–50.4%; *I*^2^ = 98.3%) and 47.9% for anxiety (95% CI: 40.9–54.9%; *I*^2^ = 98.2%). Re-including all quality-appraised reports produced pooled estimates of 44.6% for depression (95% CI: 39.6–49.6%; 66 reports) and 48.1% for anxiety (95% CI: 41.0–55.3%; 53 reports), absolute changes of 0.27 and 0.24 percentage points, respectively. Publication year was not a significant moderator for either outcome.

**Conclusion:**

Depression and anxiety symptoms were frequently reported, but estimates varied markedly across populations and instruments. The pooled estimates should be interpreted as descriptive summaries rather than precise universal prevalence values. Study-defined severity estimates and instrument-specific results may inform screening, but direct cross-instrument comparisons require caution.

## 1 Introduction

Breast cancer remains one of the most prevalent malignancies among women worldwide. In 2022, the World Health Organization (WHO) reported an estimated 2.3 million new cases, accounting for 11.5% of all female cancers. That same year, breast cancer was responsible for approximately 666,000 deaths globally, representing 6.8% of all cancer-related mortality (Ferlay et al., 2018). Advances in imaging, targeted therapy, and immunotherapy have improved early detection and treatment, raising the 5-year survival rate to nearly 90% (Miller et al., 2022). However, cancer treatment continues to pose considerable physical and psychological challenges. Surgical procedures, chemotherapy, and radiotherapy can significantly disrupt daily functioning, leading to body image concerns (e.g., breast loss or hair loss), sexual dysfunction, and persistent fatigue. These effects impair quality of life and increase the risk of social withdrawal, depression, anxiety, and other psychological disorders (Cernikova et al., 2024; Willers et al., 2025; D’Souza et al., 2017). Cancer-related stress, fear of mortality, and biological factors, such as hormonal instability and persistent symptoms, may further exacerbate the risk of developing psychological disorders (Thakur et al., 2021; Bower, 2007). Despite widespread evidence of psychological distress in breast cancer patients (Leo et al., 2024), clinical recognition and intervention remain limited, with inconsistent prevalence rates and a lack of standardized data (Gouveia et al., 2025; Qureshi et al., 2025).

Depression and anxiety are clinically important concerns in breast cancer care and have been associated with treatment adherence, postoperative pain, recurrence, and mortality. Existing syntheses have examined preoperative depression, the psychological impact of receiving a breast cancer diagnosis, or depression and anxiety in Chinese breast cancer populations. The present review extends this literature by synthesizing recent international evidence from six databases and by reporting severity-stratified and instrument-specific prevalence estimates. Its incremental contribution is therefore the joint evaluation of depression and anxiety across multiple regions and clinical contexts, with explicit attention to study-defined symptom severity and measurement instruments (Leo et al., 2024; Tao et al., 2023; Fortin et al., 2021).

This study aimed to assess the pooled prevalence of depression and anxiety in breast cancer patients and to synthesize eligible observational evidence published from January 2015 through September 10, 2026 in accordance with PRISMA guidelines.

## 2 Methods

### 2.1 Search strategy

To examine the prevalence of depression and anxiety among patients with breast cancer, we searched PubMed, Web of Science, Embase, the Cochrane Library, APA PsycINFO, and Scopus for studies published from January 1, 2015 through September 10, 2026. The database searches yielded 2,156 records from PubMed, 4,027 from Web of Science, 3,325 from Embase, 1,485 from the Cochrane Library, 97 from APA PsycINFO, and 118 from Scopus (11,208 records in total). Controlled vocabulary and free-text terms represented breast cancer, depression/anxiety, and prevalence-related concepts; the complete database-specific strategies are reported in Supplementary Table S1. Studies in all languages were eligible. All reports included in the final meta-analysis were published in English, so translation was not required. Grey literature sources and clinical trial registries were not searched.

### 2.2 Inclusion and exclusion criteria

Inclusion criteria were as follows:

Population: Clinical studies or follow-up surveys involving breast cancer patients.
Assessment Tools: Use of standardized, validated scales to assess depression and anxiety.
Publication Date: Studies published from January 2015 through September 10, 2026.
Outcomes: Reported prevalence rates of depression and/or anxiety, or sufficient data to calculate them.

#### 2.2.1 Study design

Eligible designs included cross-sectional studies and cohort studies reporting an eligible prevalence time point. Case–control studies were included only when the breast cancer group was not selected according to depression or anxiety status and an eligible breast cancer-group numerator and denominator could be extracted; control-group data were not used to estimate prevalence. Prespecified quality thresholds were applied: >17/20 for cross-sectional reports assessed with the adapted 10-domain JBI-informed rubric and >7/9 for case–control or cohort reports assessed with the Newcastle–Ottawa Scale.

#### 2.2.2 Exclusion criteria

*Non-original research*: Animal studies, study registrations, retractions, case reports, clinical guidelines, letters to the editor, reviews, meta-analyses, case studies, or conference abstracts.

*Population*: Studies that explicitly enrolled populations with a prior diagnosed mental illness were excluded because the review aimed to estimate symptoms reported in breast cancer samples without a known pre-existing psychiatric diagnosis. This restriction does not establish that breast cancer caused the symptoms and was not interpreted as supporting causal attribution.

*Data availability*: Studies in which relevant data could not be extracted or calculated.

### 2.3 Literature screening and data extraction

Two authors (Z. Y. Chen and B. T. Liu) independently screened all titles and abstracts for eligibility, followed by full-text review and data extraction. Any discrepancies were resolved through discussion with the first author (C. Y. Jia). The extracted data were subsequently verified and proofread by other members of the research team (J. L. Bai et al.).

For each potentially eligible report, we extracted study details, patient characteristics, sample size, outcome events and denominators, assessment instrument and threshold, and quality scores. A reconciled study-level screening and exclusion table is provided in Supplementary Table S3. The contemporaneous legacy log retained first-author identifiers and specific exclusion reasons; revision-stage dispositions were recorded without inventing missing bibliographic fields. Karibayeva et al. and Turdaliyeva et al. reported anxiety and depression, respectively, from the same Kazakhstan cohort; the cohort was counted once in the independent-participant total and each report contributed only its distinct outcome.

Depression and anxiety were analyzed separately. Severity analyses were limited to explicitly reported mild, moderate, and severe categories based on the validated, instrument-specific thresholds used in each source study. Overall-threshold prevalence estimates were not reclassified as a separate severity category. Because instruments differ in item content, thresholds, and reference periods, the pooled severity labels are study-defined and are not assumed to represent clinically equivalent categories across instruments.

### 2.4 Quality assessment

Two researchers (H. W. Zhang and Q. Z. Chen) independently appraised case–control and cohort reports with the Newcastle–Ottawa Scale (NOS), which evaluates selection, comparability, and outcome/exposure assessment on a 0–9 scale. The prespecified primary-analysis threshold was >7/9. Cross-sectional reports were appraised with an adapted 10-domain JBI-informed rubric rather than an unmodified standard JBI checklist. The domains covered objective/rationale, sampling frame and method, eligibility criteria, sample characteristics, measurement validity and reliability, standardized/verified data collection, ethical approval, statistical methods, results/inference, and interpretation/study value. Each domain was scored 0 (absent or major concern), 1 (partially reported or partly adequate), or 2 (clearly reported and adequate), yielding a total of 0–20. The prespecified threshold was >17/20; therefore, scores of 0–17 were excluded from the primary meta-analysis. Full scoring rules and report-level results are provided in Supplementary Table S4.

Scoring discrepancies were resolved through discussion, with a third researcher (C. Y. Jia) acting as arbiter. Final ratings and primary-analysis decisions were then reviewed and confirmed by the same researcher. The original item scores were retained; only the description of the cross-sectional framework was corrected for precision.

Inter-rater agreement in quantitative scoring is primarily ensured by standardized criteria.

### 2.5 Statistical analysis

Analyses used the final unified study-level event counts and denominators and were performed in R version 4.4.0 with the meta and metafor packages. Single proportions were transformed using the Freeman-Tukey double-arcsine transformation and pooled with a DerSimonian-Laird random-effects model before back-transformation. Heterogeneity was evaluated with Cochran’s Q, I2, and tau2. Leave-one-out analyses assessed study influence. Prespecified subgroup analyses examined geographic region, assessment instrument, and study design. A univariable random-effects meta-regression examined publication year; cancer-stage distribution and inpatient/outpatient setting were not modeled because reporting was incomplete. Funnel plots were supplemented by Egger’s regression and Begg’s rank-correlation tests when at least 10 studies were available. Because extreme heterogeneity may cause funnel-plot asymmetry unrelated to publication bias, these tests were interpreted as exploratory. A sensitivity analysis excluded all case–control studies from the depression synthesis. To evaluate the prespecified quality-based exclusion rule, we repeated the overall depression and anxiety syntheses after re-including all otherwise eligible quality-appraised reports with extractable event counts and denominators. For longitudinal reports, only pretreatment or baseline observations were used to avoid repeated contribution from the same cohort.

### 2.6 Registration

The study was registered in PROSPERO (Registration Number: CRD420251051932), and the systematic review was conducted in accordance with PRISMA guidelines.

## 3 Results

### 3.1 Literature screening

The six database searches identified 11,208 records. After 4,112 duplicates were removed, 7,096 records underwent title and abstract screening and 6,955 were excluded. Of 141 full-text reports assessed, 72 did not advance to quality appraisal. Sixty-nine reports underwent quality appraisal; 17 did not exceed the prespecified thresholds, and 52 entered the primary meta-analysis (Figure 1; Supplementary Tables S3, S4).

**Figure 1:**
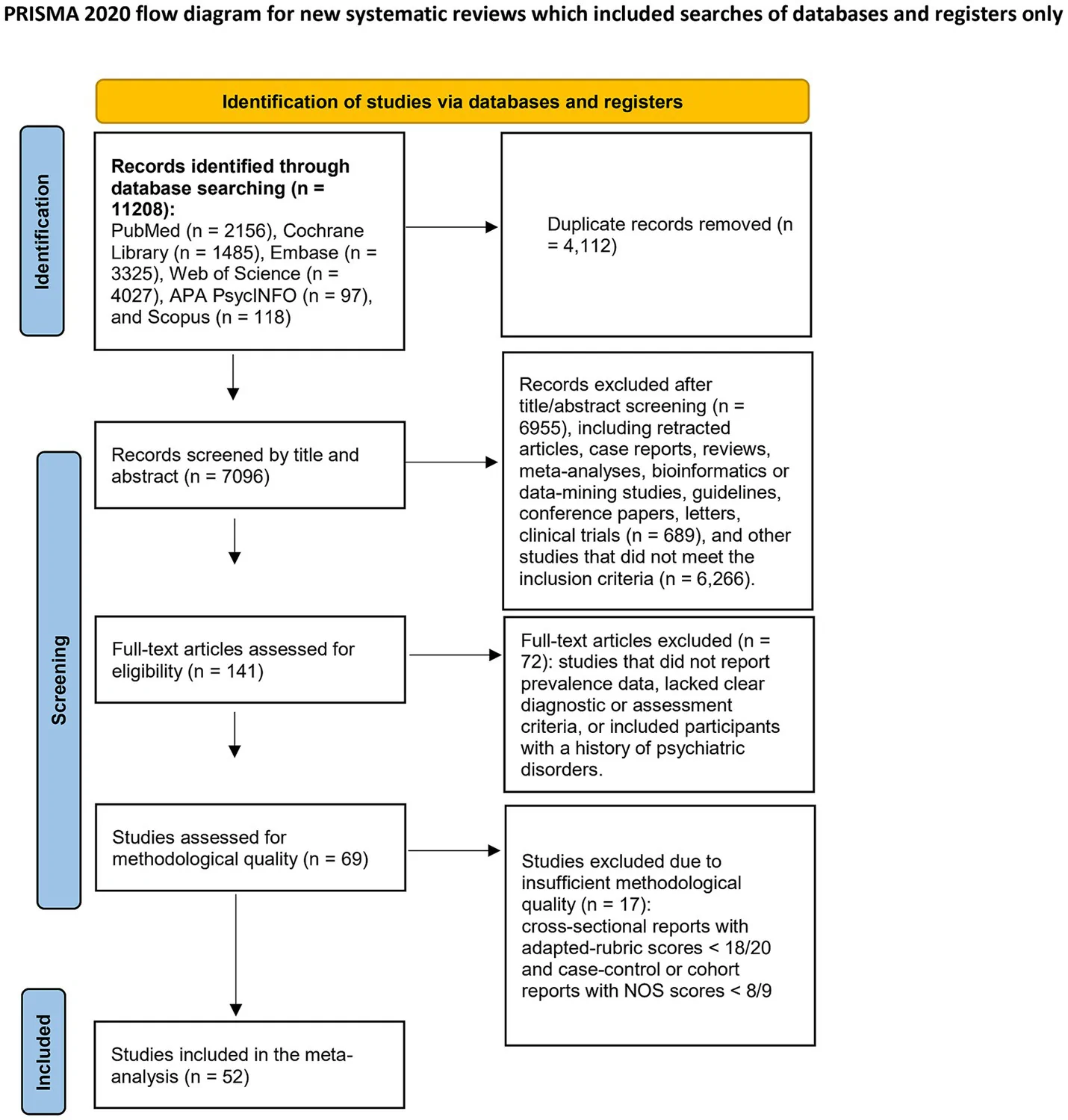
PRISMA flow diagram of study identification, screening, quality appraisal, and inclusion. *Consider, if feasible to do so, reporting the number of records identified from each database or register searched (rather than the total number across all database/registers). **If automation tools were used, indicate how many records were excluded by a human and how many were excluded by automation tools.

### 3.2 Basic characteristics of the literature

The primary meta-analysis included 52 reports: 48 cross-sectional studies, three case–control studies, and one cohort study, representing 16,850 independent participants from 27 countries. Only eligible breast cancer-group prevalence data were extracted from case–control reports. The depression synthesis included 49 reports and 16,504 participants; the anxiety synthesis included 39 reports and 11,536 participants. All included reports were published in English. Detailed characteristics are presented in Table 1 (Akechi et al., 2015; Li et al., 2015; Tojal and Costa, 2015; Fatiregun et al., 2016; Akel et al., 2017; Fradelos et al., 2017; Nho et al., 2018; Ju et al., 2018; Okubo et al., 2018; Puigpinós-Riera et al., 2018; Tsaras et al., 2018; Valderrama Rios and Sánchez Pedraza, 2018; Doege et al., 2020; Hajian-Tilaki and Hajian-Tilaki, 2020; Aggeli et al., 2021; Doubova and Pérez-Cuevas, 2021; Hajj et al., 2021; Liu et al., 2021; Abu-Helalah et al., 2022; Basto et al., 2022; Belay et al., 2022; Cáceres et al., 2022; Islam et al., 2022; Lake et al., 2022; Lopes et al., 2022; Okati-Aliabad et al., 2022; Sadaqa et al., 2022; Wulandari and Livana, 2022; Zeilinger et al., 2022; Zhao et al., 2022; Al-Fahdi et al., 2023; Guo et al., 2023; Kumar et al., 2023; Mahlaq et al., 2023; Botto et al., 2024; Buyuksimsek et al., 2024; Elamin et al., 2024; Joulaei et al., 2024; Al Alawi et al., 2025; Karibayeva et al., 2022; Civilotti et al., 2020; Park et al., 2018; Li et al., 2021; Alagizy et al., 2020; Turdaliyeva et al., 2022; Mao et al., 2025; Msenga et al., 2025; Hessami et al., 2025; Sharif et al., 2026; Gökmen et al., 2025; Al Harrasi et al., 2025; Wang et al., 2026).

**Table 1:** Characteristics of the included studies.

First author/year	Country	Study design	Sample size (n)	Population	Age (years)	Outcome	Quality score
Okubo et al. (2018)	Japan	Cross-sectional	126	Invasive breast cancer survivors diagnosed more than 1 year previously and not undergoing chemotherapy	59±11	Depression	18 (20)
Al-Fahdi et al. (2023)	Oman	Cross-sectional	171	Patients with breast cancer	50.3±10.9	Anxiety and depression	19 (20)
Guo et al. (2023)	China	Cross-sectional	176	Patients with metastatic or relapsed breast cancer undergoing chemotherapy	51.70± 9.25	Anxiety and depression	20 (20)
Kumar et al. (2023)	India	Cross-sectional	244	Patients with breast cancer	45.06±11.17	Anxiety and depression	19 (20)
Mahlaq et al. (2023)	Morocco	Cross-sectional	230	Patients with breast cancer receiving treatment for at least 8 weeks	49.2± 10.5	Anxiety and depression	18 (20)
Buyuksimsek et al. (2024)	Turkey	Cross-sectional	183	Patients with non-metastatic breast cancer	50.2± 10.75(25-75)	Depression	18 (20)
Joulaei et al. (2024)	Iran	Cross-sectional	311	Patients with breast cancer	52.26±9.66	Anxiety and depression	19 (20)
Al Alawi et al. (2025)	Oman	Cross-sectional	105	Patients with breast cancer; stage IV disease excluded	43.6±7.2	Anxiety and depression	19 (20)
Li et al. (2015)	China	Case-control	247	Women with early breast cancer	47.45± 7.43	Depression	8 (9)
Doege et al. (2020)	Germany	Case-control	3010	Long-term breast cancer survivors	30-89	Depression	8 (9)
Puigpinós-Riera et al. (2018)	Spain	Cross-sectional	2235	Women with breast cancer	<50, 50-65, >65	Anxiety and depression	19 (20)
Lopes et al. (2022)	Portugal	Cohort	506	Women with newly diagnosed breast cancer scheduled for surgery	<55 or ≥55	Anxiety and depression	8 (9)
Elamin et al. (2024)	Saudi Arabia	Cross-sectional	56	Women scheduled to receive radiotherapy for non-metastatic stage I-III breast cancer	50.1± 11.94(28-79)	Anxiety and depression	19 (20)
Botto et al. (2024)	Italy	Cross-sectional	301	Women diagnosed with stage I-III breast cancer	≥18	Anxiety and depression	18 (20)
Islam et al. (2022)	Bangladesh	Cross-sectional	95	Patients with metastatic breast cancer receiving palliative care	48.9± 9.9	Anxiety and depression	18 (20)
Lake et al. (2022)	USA	Cross-sectional	351	Black breast cancer survivors diagnosed at or before 50 years of age	44	Anxiety and depression	18 (20)
Okati-Aliabad et al. (2022)	Iran	Cross-sectional	120	Women with breast cancer attending oncology or radiotherapy departments	47.35± 10.67	Anxiety and depression	18 (20)
Sadaqa et al. (2022)	Palestine	Cross-sectional	233	Women aged 30-70 years with primary breast cancer diagnosed within the previous year	30-70	Depression	18 (20)
Wulandari and Livana (2022)	Indonesia	Cross-sectional	102	Patients with breast cancer receiving outpatient or inpatient oncology care	>20	Depression	18 (20)
Zeilinger et al. (2022)	Austria	Cross-sectional	1194	Women with breast cancer	58.9±14	Anxiety and depression	18 (20)
Zhao et al. (2022)	China	Cross-sectional	398	Patients with breast cancer treated at Shanxi Bethune Hospital	24-92	Anxiety and depression	18 (20)
Hajian-Tilaki and Hajian-Tilaki (2020)	Iran	Cross-sectional	305	Breast cancer survivors	49.5±10.1	Anxiety and depression	18 (20)
Aggeli et al. (2021)	Greece	Cross-sectional	170	Breast cancer survivors	61-70	Anxiety and depression	18 (20)
Doubova and Pérez-Cuevas (2021)	Mexico	Cross-sectional	247	Women with breast or cervical cancer	≥46	Depression	18 (20)
Hajj et al. (2021)	Lebanon	Cross-sectional	112	Women with primary breast cancer receiving chemotherapy	56.04± 11.69	Anxiety and depression	19 (20)
Liu et al. (2021)	China	Cross-sectional	389	Patients with breast cancer	≥18	Anxiety and depression	18 (20)
Abu-Helalah et al. (2022)	Saudi Arabia	Cross-sectional	246	Women with breast cancer diagnosed 6-12 months previously	49.5±10.9	Anxiety and depression	19 (20)
Basto et al. (2022)	Portugal	Cross-sectional	91	Women with metastatic breast cancer receiving palliative treatment	59.79	Anxiety and depression	19 (20)
Belay et al. (2022)	Ethiopia	Cross-sectional	333	Patients with breast cancer	≥18	Anxiety and depression	18 (20)
Cáceres et al. (2022)	Spain	Cross-sectional	200	Women with breast cancer receiving oncological treatment	53.05± 10.71	Depression	18 (20)
Tsaras et al. (2018)	Greece	Cross-sectional	152	Breast cancer survivors	53.25±12.10	Anxiety and depression	18 (20)
Valderrama Rios and Sánchez Pedraza (2018)	Colombia	Cross-sectional	107	Patients with locally advanced or metastatic breast cancer	53.9± 8.7	Anxiety and depression	19 (20)
Akel et al. (2017)	USA	Cross-sectional	150	Women diagnosed with breast cancer at least 6 months previously	53.5±10.4	Anxiety and depression	18 (20)
Fradelos et al. (2017)	Greece	Cross-sectional	144	Breast cancer survivors	53.5±11.7	Anxiety and depression	19 (20)
Ju et al. (2018)	Korea	Cross-sectional	198	Women who had undergone surgery for breast cancer	53.1±10.3	Anxiety and depression	19 (20)
Nho et al. (2018)	Korea	Cross-sectional	241	Breast cancer survivors	46.89±8.74	Anxiety and depression	18 (20)
Akechi et al. (2015)	Japan	Cross-sectional	146	Long-term disease-free breast cancer survivors	57±11	Anxiety	20 (20)
Fatiregun et al. (2016)	Nigeria	Cross-sectional	200	Women attending an oncology outpatient clinic with breast cancer	49.6±11.2	Anxiety	20 (20)
Tojal and Costa (2015)	Portugal	Cross-sectional	150	Portuguese-speaking women diagnosed with breast cancer	<40, 40-50, >50	Depression	18 (20)
Civilotti et al. (2020)	Italy	Cross-sectional	436	Women newly diagnosed with breast cancer and awaiting surgery	57.18±12.48	Anxiety and depression	19 (20)
Park et al. (2018)	USA	Cross-sectional	54	Young women with metastatic breast cancer	≤40	Anxiety and depression	18 (20)
Li et al. (2021)	China	Case-control	120	Women with breast cancer; breast cancer group used for prevalence	40.85±6.50	Anxiety and depression	8 (9)
Alagizy et al. (2020)	Egypt	Cross-sectional	64	Patients with breast cancer at a single institute	52.29±11.64	Anxiety and depression	18 (20)
Karibayeva et al. (2022)	Kazakhstan	Cross-sectional	162	Women newly diagnosed with breast cancer	54.41±8.10	Anxiety	18 (20)
Turdaliyeva et al. (2022)	Kazakhstan	Cross-sectional	162	Women newly diagnosed with breast cancer; same cohort as Karibayeva	54.41±8.10	Depression	18 (20)
Mao et al. (2025)	China	Multicenter cross-sectional	360	Middle-aged and young patients with breast cancer	NR	Depression	18 (20)
Msenga et al. (2025)	Tanzania	Cross-sectional	384	Women diagnosed with breast cancer at a national cancer institute	NR	Anxiety and depression	19 (20)
Hessami et al. (2025)	Iran	Cross-sectional	283	Patients with breast cancer	NR	Anxiety and depression	19 (20)
Sharif et al. (2026)	Pakistan	Cross-sectional	96	Breast cancer patients receiving treatment at two clinical centers	NR	Anxiety and depression	18 (20)
Gökmen et al. (2025)	Türkiye	Multicenter cross-sectional	460	Patients with breast cancer assessed by interview and medical records	NR	Anxiety and depression	19 (20)
Al Harrasi et al. (2025)	Oman	Cross-sectional	154	Adult Omani female breast cancer survivors	41.7±6.7	Depression	19 (20)
Wang et al. (2026)	China	Cross-sectional	302	Patients with breast cancer in a dietary antioxidant study	NR	Depression	18 (20)

BC, breast cancer; CT, chemotherapy; JBI, Joanna Briggs Institute; NOS, Newcastle-Ottawa Scale. Quality scores are reported as obtained score (maximum possible score). Cross-sectional studies were assessed using the JBI checklist; case-control and cohort studies were assessed using NOS.

Among the 52 included reports, 49 contributed depression data and 39 contributed anxiety data. Explicit mild, moderate, and severe depression data were available from 16, 21, and 14 reports, respectively. Explicit mild, moderate, and severe anxiety data were available from 12, 16, and 11 reports, respectively. These severity syntheses used study-defined instrument thresholds; studies reporting only an overall threshold contributed to the overall prevalence analysis but not to a severity category.

### 3.3 Summary results of depression prevalence

Forty-nine reports involving 16,504 participants contributed to the depression analysis. The random-effects pooled prevalence was 44.3% (95% CI: 38.3–50.4%), with extreme heterogeneity (*I*^2^ = 98.3%; tau2 = 0.0456; Figure 2a). By instrument, pooled prevalence was 64.4% for BDI (95% CI: 35.8–88.4%), 47.9% for DASS-21-D (95% CI: 41.1–54.7%), 39.8% for HADS-D (95% CI: 30.7–49.4%), 46.1% for two-item scales (95% CI: 30.8–61.8%), and 45.6% for other tools (95% CI: 36.1–55.3%); the between-tool difference was not significant (*p* = 0.479; Supplementary Table S2). Publication year was not a significant moderator (*p* = 0.946).

**Figure 2:**
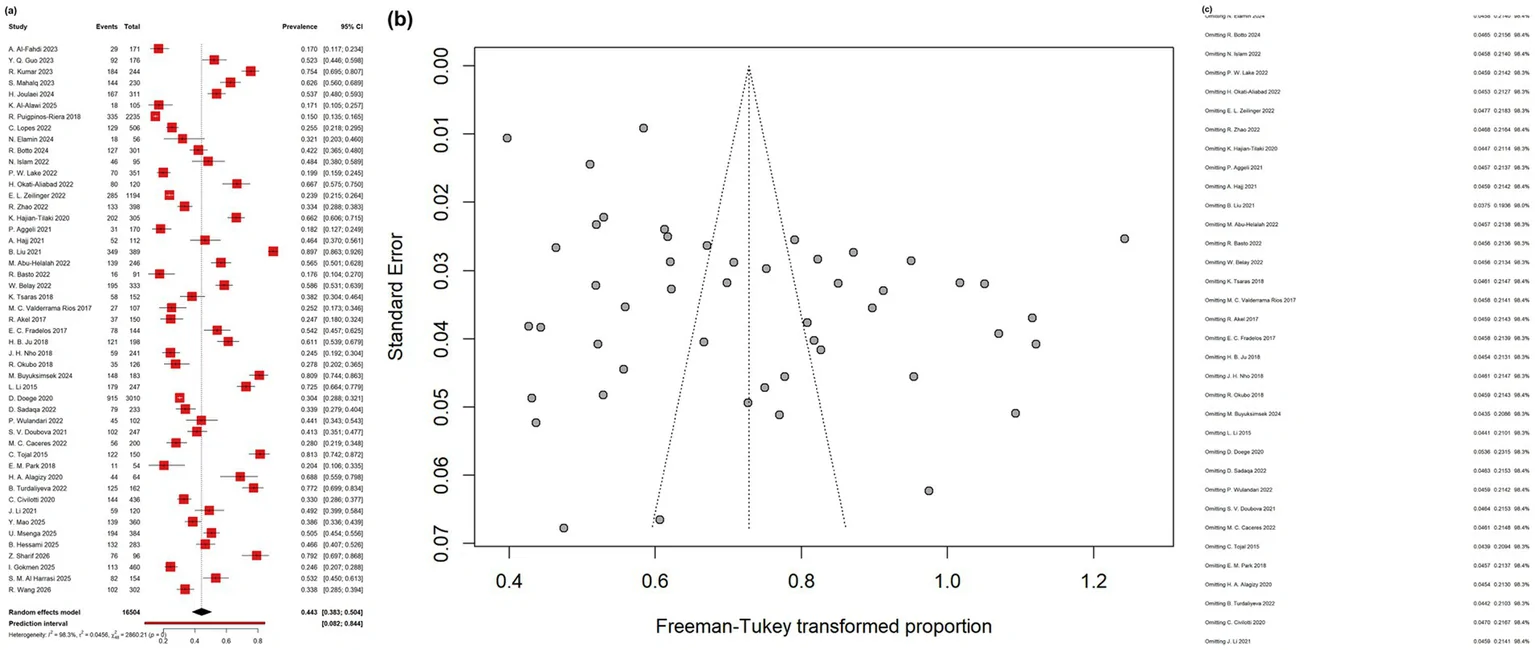
The summary results of the prevalence of depression. **(a)** Forest plot; **(b)** Funnel chart; **(c)** Sensitivity analysis.

The depression funnel plot showed some asymmetry, but neither Egger’s regression (*p* = 0.523) nor Begg’s rank-correlation test (*p* = 0.330) was statistically significant. In view of the extreme heterogeneity and the absence of a grey-literature search, these findings do not exclude reporting bias or small-study effects (Figure 2b).

Leave-one-out analysis did not identify a single study that dominated the depression estimate (Figure 2c). Excluding the three case–control studies left 46 reports and produced a similar pooled prevalence of 43.9% (95% CI: 37.3–50.6%; *I*^2^ = 98.3%), indicating that study design did not materially account for the overall estimate or heterogeneity.

#### 3.3.1 Mild depression

Sixteen reports involving 5,774 participants provided an explicit mild-depression category. The pooled prevalence was 20.2% (95% CI: 16.0–24.9%; *I*^2^ = 92.4%; Supplementary Figure S1). The label reflects each study’s instrument-specific cutoff and should not be interpreted as a common diagnostic threshold.

#### 3.3.2 Moderate depression

Twenty-one reports involving 4,328 participants provided an explicit moderate-depression category. The pooled prevalence was 28.4% (95% CI: 23.1–33.9%; *I*^2^ = 93.6%; Supplementary Figure S2).

#### 3.3.3 Severe depression

Fourteen reports involving 9,572 participants provided an explicit severe-depression category. The pooled prevalence was 16.7% (95% CI: 12.0–22.1%; *I*^2^ = 97.4%; Supplementary Figure S3).

### 3.4 Summary results of anxiety prevalence

Thirty-nine reports involving 11,536 participants contributed to the anxiety analysis. The random-effects pooled prevalence was 47.9% (95% CI: 40.9–54.9%), with extreme heterogeneity (*I*^2^ = 98.2%; tau2 = 0.0490; Figure 3a). By instrument, pooled prevalence was 54.8% for DASS-21-A (95% CI: 46.8–62.6%), 52.1% for HADS-A (95% CI: 43.6–60.4%), 39.2% for two-item scales (95% CI: 25.8–53.5%), and 37.9% for other tools (95% CI: 23.7–53.2%); the between-tool difference was not significant (*p* = 0.102; Supplementary Table S2). Publication year was not a significant moderator (*p* = 0.649).

**Figure 3:**
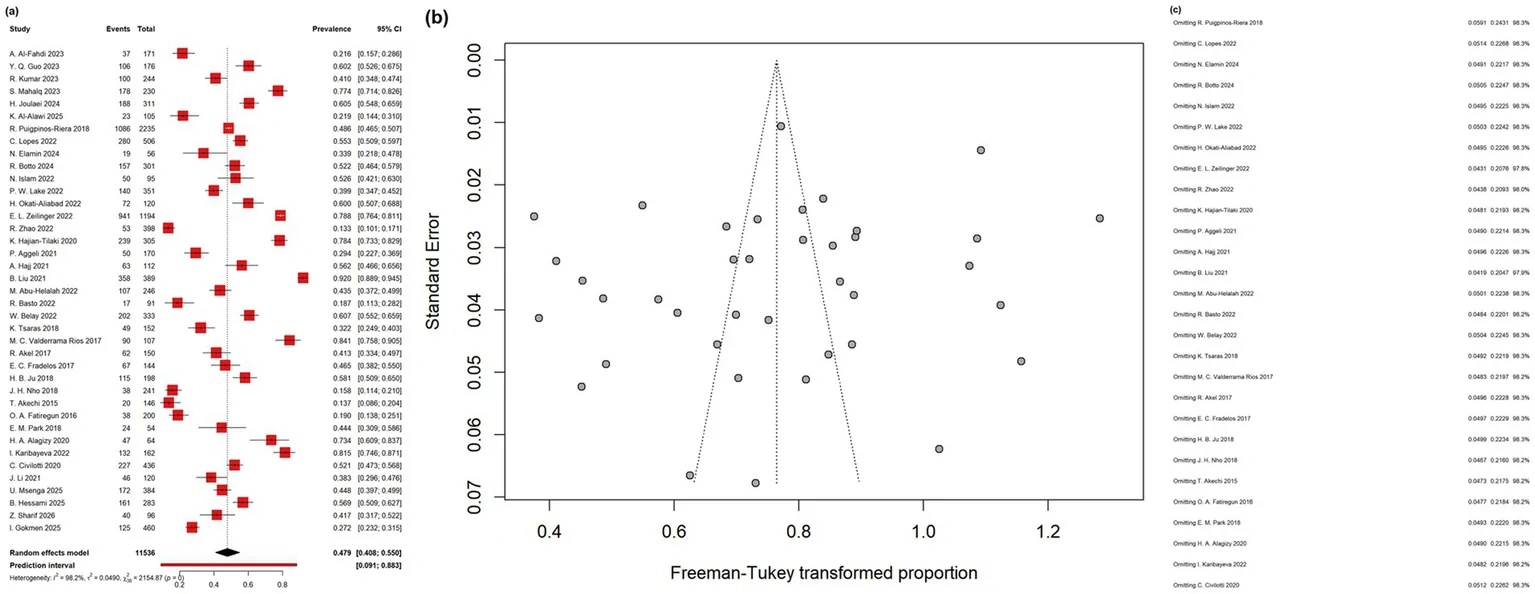
The summary results of anxiety disorder prevalence. **(a)** Forest plot; **(b)** Funnel chart; **(c)** Sensitivity analysis.

The anxiety funnel plot showed some asymmetry, but neither Egger’s regression (*p* = 0.420) nor Begg’s rank-correlation test (*p* = 0.663) was statistically significant. Extreme heterogeneity limits the ability of these tests to rule out reporting bias or small-study effects (Figure 3b).

Leave-one-out analysis did not identify a single study that dominated the anxiety estimate; the extreme heterogeneity remained (Figure 3c).

#### 3.4.1 Mild anxiety

Twelve reports involving 3,355 participants provided an explicit mild-anxiety category. The pooled prevalence was 16.7% (95% CI: 12.6–21.3%; *I*^2^ = 90.1%; Supplementary Figure S4). The label reflects study-specific instrument thresholds.

#### 3.4.2 Moderate anxiety

Sixteen reports involving 2,946 participants provided an explicit moderate-anxiety category. The pooled prevalence was 30.7% (95% CI: 23.9–38.0%; *I*^2^ = 94.3%; Supplementary Figure S5).

#### 3.4.3 Severe anxiety

Eleven reports involving 3,427 participants provided an explicit severe-anxiety category. The pooled prevalence was 18.2% (95% CI: 7.4–32.6%; *I*^2^ = 98.9%; Supplementary Figure S6).

### 3.5 Quality-threshold sensitivity analysis

All 69 quality-appraised reports were considered in the quality-threshold sensitivity analysis; reports contributed to an outcome only when an eligible numerator and denominator were available. For depression, re-including the 17 quality-excluded reports increased the synthesis from 49 reports (16,504 participants) to 66 reports (31,462 participants). The pooled prevalence was 44.6% (95% CI: 39.6–49.6%; *I*^2^ = 98.6%; tau2 = 0.0416), compared with 44.3% in the primary analysis, an absolute change of 0.27 percentage points. For anxiety, re-including 14 quality-excluded reports increased the synthesis from 39 reports (11,536 participants) to 53 reports (25,751 participants). The pooled prevalence was 48.1% (95% CI: 41.0–55.3%; *I*^2^ = 99.1%; tau2 = 0.0699), compared with 47.9% in the primary analysis, an absolute change of 0.24 percentage points. Thus, the principal estimates were robust to the quality-based exclusion rule, although heterogeneity remained extreme (Supplementary Table S5; Supplementary Figures S9–S11).

## 4 Discussion

This meta-analysis included 52 reports representing 16,850 independent participants. The pooled prevalence was 44.3% for depression and 47.9% for anxiety, but I2 values exceeded 98%, and substantial heterogeneity persisted within most subgroups. These percentages are descriptive summaries of diverse study populations and measurement approaches, not precise universal prevalence parameters. Publication year was not a significant moderator for either outcome. Region and study design showed significant subgroup differences, whereas between-instrument differences were not statistically significant; sparse strata and residual heterogeneity limit causal interpretation of subgroup findings. The quality-threshold sensitivity analysis yielded nearly identical pooled estimates after all otherwise eligible quality-appraised reports were re-included (44.6% for depression and 48.1% for anxiety), supporting the robustness of the principal estimates to the prespecified score-based exclusion rule while confirming that heterogeneity remained extreme.

Severity-stratified analyses were restricted to explicitly reported mild, moderate, and severe categories. Moderate categories had the highest pooled estimates for both depression and anxiety, but the categories came from different subsets of studies and instruments with different thresholds and reference periods. They should not be summed into a single distribution or treated as clinically equivalent across tools. The findings support validated psychological screening in breast cancer care while leaving instrument selection and referral thresholds to clinical context and established guidance.

The relatively high prevalence of psychological disorders reported in breast cancer patients may be related to a complex interaction of physical, psychological, social, and cultural factors. The diagnosis itself is a major psychological shock, often triggering acute stress reactions such as anxiety and adjustment disorders, with 31% of patients experiencing such symptoms early in diagnosis (Fortin et al., 2021). Advanced cancer stages are associated with more severe depression and anxiety symptoms (Abu-Odah et al., 2022). A persistent fear of recurrence, common even post-treatment, is strongly linked to depressive symptoms (Schapira et al., 2022). Treatment-related decisions, such as choosing between lumpectomy and mastectomy, can cause decision fatigue, weakening psychological resilience and increasing anxiety (Silverstein et al., 2023). Side effects like chemotherapy-induced hair loss often lead to body image concerns and prolonged low mood (Choi et al., 2014).

A postoperative survey from King Abdulaziz University showed that 57.78% of respondents reported psychological issues, primarily due to altered body image, while 26.67% experienced distress from disfiguring scars, and 56.48% reported social withdrawal (Barkar et al., 2023). Sexual dysfunction (e.g., vaginal dryness, decreased libido) was reported by 31.6% of younger patients and significantly correlated with depression (Lee et al., 2015). Biological factors, including hormonal fluctuations and disease symptoms, also contribute to psychological distress.

Inadequate social support and financial hardship further exacerbate mental health challenges. Limited family involvement, emotional withdrawal by partners, and caregiving burdens contribute to isolation. A social support assessment showed that depressed patients receiving support from significant others had a mean score of 190.11 versus 221.99 in non-depressed patients, highlighting a negative correlation between support and depression (Wondimagegnehu et al., 2019). Financial strain may lead to care interruptions or pursuit of unstandardized therapies. A multicenter study found a significant association between economic toxicity and depression (*r* = −0.11, *p* < 0.05) (Chen et al., 2024). In cultures with strong family values, role reversal from caregiver to dependent often lowers self-esteem and fosters helplessness (Cobo-Cuenca et al., 2019).

Cultural context also plays a key role in how psychological symptoms manifest and are reported. For instance, 81% of breast cancer patients in Kazakhstan reported moderate to severe anxiety, compared to 30.8% in South Korea, possibly reflecting differences in collectivist cultural norms (Karibayeva et al., 2022; Cho and Hwang, 2021). Religious or traditional beliefs, such as attributing illness to divine will, may further intensify emotional distress. These factors can, in turn, negatively affect treatment adherence (Gall and Bilodeau, 2020).

Depression and anxiety appear to be common in breast cancer patients, and their potential impact on treatment outcomes may vary according to symptom severity and duration. This study emphasized prevalence by symptom severity, as both conditions significantly affect quality of life, treatment adherence, and survival (So et al., 2010). Clinically, their importance is often underestimated, leading to impaired communication and reduced empathy toward patients (Broadbridge and Venetis, 2024). The gap between physician-diagnosed and self-reported cases may stem from inadequate implementation of clinical guidelines and variation in assessment tools (Mitchell et al., 2009). Since no objective biomarkers exist, depression and anxiety are typically assessed via patient-reported questionnaires. Our findings support the clinical relevance of depression and anxiety in this population and suggest that mental health management may be an important component of breast cancer care. Routine psychological assessment and follow-up could be considered, particularly at diagnosis, during major treatment phases, and in survivorship follow-up. For patients with mild to moderate symptoms, cognitive-behavioral therapy or structured group interventions, together with physical activity, may be helpful (Yan et al., 2025; Novakov et al., 2019). Patients with more severe symptoms may benefit from timely referral for specialist psychiatric evaluation (Lee and Lee, 2020). Multidisciplinary collaboration between oncology and mental health services may improve the delivery and acceptability of supportive care (Probst et al., 2025).

This review has several limitations. First, heterogeneity remained extreme despite subgroup analyses, so pooled values summarize clinically and methodologically diverse studies. Second, most reports were cross-sectional and cannot support causal inference. Third, severity labels were based on study-specific instruments and thresholds and are not clinically equivalent across tools. Fourth, excluding populations with a prior mental illness may underestimate the psychological burden encountered in routine oncology practice. Fifth, grey literature and trial registries were not searched. Sixth, cancer stage, treatment phase, and inpatient/outpatient setting were too incompletely reported for meta-regression. Seventh, the cross-sectional quality appraisal used an adapted 10-domain JBI-informed scoring rubric rather than an unmodified standard JBI checklist; the scoring rules and item-level results are now fully disclosed in Supplementary Table S4. Finally, case–control reports contributed only eligible breast cancer-group prevalence data; the sensitivity analysis excluding them produced a similar depression estimate but did not reduce heterogeneity. Although re-including the quality-excluded reports yielded nearly identical pooled estimates, this robustness check does not validate the customized score threshold as a universal standard.

## 5 Conclusion

Depression and anxiety symptoms were frequently reported in breast cancer populations, with pooled descriptive estimates of 44.3 and 47.9%, respectively. Marked between-study heterogeneity and variation in instruments limit the precision and transferability of these values. Study-defined severity results may help frame screening and referral, but cross-instrument comparisons require caution. Future multicenter prospective studies should use clearly reported sampling methods, validated thresholds, and standardized descriptions of disease and treatment stage.

## Data Availability

The original contributions presented in the study are included in the article/Supplementary material, further inquiries can be directed to the corresponding author/s.

## References

[ref1] Abu-Helalah M. Mustafa H. Alshraideh H. Alsuhail A. I. Almousily O. A. Al-Abdallah R. . (2022). Quality of life and psychological wellbeing of breast cancer survivors in the Kingdom of Saudi Arabia. Asian Pac. J. Cancer Prev. 23, 2291–2297. doi: 10.31557/APJCP.2022.23.7.2291, 35901334 PMC9727347

[ref2] Abu-Odah H. Molassiotis A. Zhao I. Y. Su J. J. Allsop M. J. (2022). Psychological distress and associated factors among Palestinian advanced cancer patients: a cross-sectional study. Front. Psychol. 13:1061327. doi: 10.3389/fpsyg.2022.1061327, 36533049 PMC9755485

[ref3] Aggeli P. Fasoi G. Zartaloudi A. Kontzoglou K. Kontos M. Konstantinidis T. . (2021). Posttreatment anxiety, depression, sleep disorders, and associated factors in women who survive breast Cancer. Asia Pac. J. Oncol. Nurs. 8, 147–155. doi: 10.4103/apjon.apjon_65_20, 33688563 PMC7934591

[ref4] Akechi T. Momino K. Miyashita M. Sakamoto N. Yamashita H. Toyama T. (2015). Anxiety in disease-free breast cancer patients might be alleviated by provision of psychological support, not of information. Jpn. J. Clin. Oncol. 45, 929–933. doi: 10.1093/jjco/hyv112, 26185138

[ref5] Akel R. El Darsa H. Anouti B. Mukherji D. Temraz S. Raslan R. . (2017). Anxiety, depression and quality of life in breast cancer patients in the levant. Asian Pac. J. Cancer Prev. 18, 2809–2816. doi: 10.22034/APJCP.2017.18.10.2809, 29072421 PMC5747408

[ref6] Al Alawi K. Al Fahdi A. Chan M. F. Al Sumri H. Al-Azri M. (2025). Evaluating symptom burden among Omani women newly diagnosed with breast cancer: a cross-sectional study. Current oncology (Toronto, Ont.) 32:59. doi: 10.3390/curroncol32020059, 39996859 PMC11854252

[ref7] Al Harrasi S. M. Al-Awaisi H. Al Balushi M. Al Balushi L. Al Kharusi S. (2025). Fear of cancer recurrence and its association with depressive symptoms among adult Omani female breast cancer survivors. Int. J. Women’s Health 17, 2625–2635. doi: 10.2147/IJWH.S541449, 40860640 PMC12377397

[ref8] Alagizy H. A. Soltan M. R. Soliman S. S. Hegazy N. N. Gohar S. F. (2020). Anxiety, depression and perceived stress among breast cancer patients: single institute experience. Middle East Curr. Psychiatry 27:29. doi: 10.1186/s43045-020-00036-x

[ref9] Al-Fahdi A. Chan M. F. Al-Siyabi W. Al-Yafai E. Al-Khatri M. Al-Azri M. (2023). Prevalence of psychological distress and associated factors among Omani women diagnosed with breast cancer: a single-Centre, cross-sectional study. BMJ Open 13:e073967. doi: 10.1136/bmjopen-2023-073967, 37734894 PMC10514623

[ref10] Barkar M. A. Mikwar Z. Khalid A. A. Mohammedamin A. A. Aloufi A. H. Abualhamail A. A. . (2023). Patient satisfaction and quality of life after mastectomy at king Abdulaziz Medical City, Jeddah. Cureus 15:e51029. doi: 10.7759/cureus.51029, 38149063 PMC10750441

[ref11] Basto R. Caramujo C. Ferreira Gomes I. Fraga T. Correia Magalhães J. Sousa M. J. . (2022). Screening patients with metastatic breast cancer for psychiatric disease: a cross sectional study. F1000Res 11:73. doi: 10.12688/f1000research.79400.1

[ref12] Belay W. Labisso W. L. Tigeneh W. Kaba M. Haileselassie W. (2022). Magnitude and factors associated with anxiety and depression among patients with breast cancer in Central Ethiopia: a cross-sectional study. Front. Psych. 13:957592. doi: 10.3389/fpsyt.2022.957592, 36090359 PMC9458879

[ref13] Botto R. De Leonardis B. Salussolia A. Di Nardo C. Ala A. Torta R. . (2024). Assessment of anxiety, depression, and distress in breast cancer patients: a comparison among different phases of illness. Health Care Women Int. 45, 390–402. doi: 10.1080/07399332.2022.2078325, 35695501

[ref14] Bower J. E. (2007). Cancer-related fatigue: links with inflammation in cancer patients and survivors. Brain Behav. Immun. 21, 863–871. doi: 10.1016/j.bbi.2007.03.013, 17543499 PMC3630796

[ref15] Broadbridge E. A. Venetis M. K. (2024). Examining the role of pre-visit anxiety on patient uncertainty and breast cancer patient-provider communication. Cancer Med. 13:e70003. doi: 10.1002/cam4.70003, 39031003 PMC11258471

[ref16] Buyuksimsek M. Gulmez A. Pirinci O. Tohumcuoglu M. Kidi M. M. (2024). Are depression and poor sleep quality a major problem in Turkish women receiving chemotherapy for breast cancer? Rev. Assoc. Med. Bras. (1992) 70:e20231377. doi: 10.1590/1806-9282.20231377, 38656011 PMC11042824

[ref17] Cáceres M. C. Nadal-Delgado M. López-Jurado C. Pérez-Civantos D. Guerrero-Martín J. Durán-Gómez N. (2022). Factors related to anxiety, depressive symptoms and quality of life in breast cancer. Int. J. Environ. Res. Public Health 19:3547. doi: 10.3390/ijerph19063547, 35329232 PMC8955820

[ref18] Cernikova K. A. Kracmarova L. K. Pesoutova M. Tavel P. (2024). We will be different forever: a qualitative study of changes of body image in women with breast cancer. BMC Public Health 24:2517. doi: 10.1186/s12889-024-20017-7, 39285297 PMC11403935

[ref19] Chen X. Yan Q. Tang Y. Zhu J. Zhang W. Zhang J. (2024). Financial toxicity, family resilience and negative emotions among young and middle-aged breast cancer patients: a multicentre cross-sectional study. Breast 75:103735. doi: 10.1016/j.breast.2024.103735, 38640552 PMC11031793

[ref20] Cho O. H. Hwang K. H. (2021). Association between sleep quality, anxiety and depression among Korean breast cancer survivors. Nurs. Open 8, 1030–1037. doi: 10.1002/nop2.710, 34482659 PMC8046065

[ref21] Choi E. K. Kim I. R. Chang O. Kang D. Nam S. J. Lee J. E. . (2014). Impact of chemotherapy-induced alopecia distress on body image, psychosocial well-being, and depression in breast cancer patients. Psychooncology 23, 1103–1110. doi: 10.1002/pon.3531, 24664939

[ref22] Civilotti C. Botto R. Maranesi M. Leonardis B. Bianciotto B. Stanizzo M. R. (2020). Anxiety and depression in women newly diagnosed with breast cancer and waiting for surgery: prevalence and associations with socio-demographic variables. Support Care Cancer 28, 4997–5004. doi: 10.1007/s00520-020-05343-x PMC8148592 34066935

[ref23] Cobo-Cuenca A. I. Martín-Espinosa N. M. Rodríguez-Borrego M. A. Carmona-Torres J. M. (2019). Determinants of satisfaction with life and self-esteem in women with breast cancer. Qual. Life Res. 28, 379–387. doi: 10.1007/s11136-018-2017-y, 30324585

[ref24] D’Souza V. Daudt H. Kazanjian A. (2017). Survivorship care plans for breast cancer patients: understanding the quality of the available evidence. Curr. Oncol. 24, e446–e465. doi: 10.3747/co.24.3632, 29270054 PMC5736484

[ref26] Doege D. Thong M. S. Y. Koch-Gallenkamp L. Jansen L. Bertram H. Eberle A. . (2020). Age-specific prevalence and determinants of depression in long-term breast cancer survivors compared to female population controls. Cancer Med. 9, 8713–8721. doi: 10.1002/cam4.3476, 33022889 PMC7666751

[ref27] Doubova S. V. Pérez-Cuevas R. (2021). Association of supportive care needs and quality of patient-centered cancer care with depression in women with breast and cervical cancer in Mexico. Psychooncology 30, 591–601. doi: 10.1002/pon.5608, 33247968

[ref28] Elamin N. Althebity N. Alkhamisi T. A. Al-Foheidi M. (2024). Sleep quality and psychological disorders in breast cancer female patients receiving radiotherapy at a tertiary oncology center in West Saudi Arabia. Support Care Cancer 32:163. doi: 10.1007/s00520-024-08368-8, 38367029

[ref29] Fatiregun O. A. Olagunju A. T. Erinfolami A. R. Fatiregun O. A. Arogunmati O. A. Adeyemi J. D. (2016). Anxiety disorders in breast cancer: prevalence, types, and determinants. J. Psychosoc. Oncol. 34, 432–447. doi: 10.1080/07347332.2016.1196805, 27269867

[ref30] Ferlay J. Ervik M. Lam F. Colombet M. Mery L. Piñeros M. . (2018). Global Cancer Observatory: Cancer Today. Lyon, France: International Agency for Research on Cancer.

[ref31] Fortin J. Leblanc M. Elgbeili G. Cordova M. J. Marin M. F. Brunet A. (2021). The mental health impacts of receiving a breast cancer diagnosis: a meta-analysis. Br. J. Cancer 125, 1582–1592. doi: 10.1038/s41416-021-01542-3, 34482373 PMC8608836

[ref32] Fradelos E. C. Papathanasiou I. V. Veneti A. Daglas A. Christodoulou E. Zyga S. . (2017). Psychological distress and resilience in women diagnosed with breast cancer in Greece. Asian Pac. J. Cancer Prev. 18, 2545–2550. doi: 10.22034/APJCP.2017.18.9.2545, 28952298 PMC5720664

[ref33] Gall T. L. Bilodeau C. (2020). The role of positive and negative religious/spiritual coping in women's adjustment to breast cancer: a longitudinal study. J. Psychosoc. Oncol. 38, 103–117. doi: 10.1080/07347332.2019.1641581, 31317825

[ref34] Gökmen İ. Divriklioğlu D. Özcan E. Çakici V. Coşkun A. Demir N. . (2025). Comprehensive mapping of psychosocial burden in breast cancer: a multicenter cross-sectional study in Türkiye. Namık Kemal Tıp Derg. 13, 231–245. doi: 10.4274/nkmj.galenos.2025.35492

[ref35] Gouveia M. C. Scaranti M. Migliavacca Zucchetti B. Bonadio R. C. Barroso-Sousa R. Leone J. P. (2025). Unveiling the paradigm shift: systemic treatment strategies in small, node-negative breast cancer. NPJ Breast Cancer 11:44. doi: 10.1038/s41523-025-00761-8, 40379683 PMC12084305

[ref36] Guo Y. Q. Ju Q. M. You M. Liu Y. Yusuf A. Soon L. K. (2023). Depression, anxiety and stress among metastatic breast cancer patients on chemotherapy in China. BMC Nurs. 22:33. doi: 10.1186/s12912-023-01184-1, 36747213 PMC9901155

[ref37] Hajian-Tilaki K. Hajian-Tilaki E. (2020). Factor structure and reliability of Persian version of hospital anxiety and depression scale in patients with breast cancer survivors. Health Qual. Life Outcomes 18:176. doi: 10.1186/s12955-020-01429-6, 32522209 PMC7288677

[ref38] Hajj A. Hachem R. Khoury R. Hallit S. El Jebbawi B. Nasr F. . (2021). Clinical and genetic factors associated with anxiety and depression in breast cancer patients: a cross-sectional study. BMC Cancer 21:872. doi: 10.1186/s12885-021-08615-9, 34330229 PMC8323303

[ref39] Hessami B. Hessami A. Ghadirzadeh E. Ashrafi S. Taghavi F. Elyasi F. . (2025). Depression, anxiety, and stress in breast cancer patients: prevalence, associated risk factors, and clinical correlates. Sci. Rep. 15:285. doi: 10.1038/s41598-025-17285-7, 40851057 PMC12375722

[ref40] Islam N. Biswas J. Kowshik M. M. Molla M. M. A. Sarker M. Chowdhury M. K. . (2022). Depression, anxiety, and performance status among the women with metastatic breast cancer receiving palliative care in Bangladesh: a cross sectional study. Health Sci. Rep. 5:e911. doi: 10.1002/hsr2.911, 36320658 PMC9617648

[ref41] Joulaei H. Parhizkar M. Fatemi M. Afrashteh S. Parhizkar P. Akrami M. . (2024). Mental health care utilization and its barriers among Iranian breast cancer survivors: a cross-sectional study. Int. J. Community Based Nurs. Midwifery 12, 44–56. doi: 10.30476/IJCBNM.2023.99133.2289, 38328011 PMC10844875

[ref42] Ju H. B. Kang E. C. Jeon D. W. Kim T. H. Moon J. J. Kim S. J. . (2018). Associations among plasma stress markers and symptoms of anxiety and depression in patients with breast Cancer following surgery. Psychiatry Investig. 15, 133–140. doi: 10.30773/pi.2017.07.26, 29475233 PMC5900404

[ref43] Karibayeva I. Turdaliyeva B. Zainal N. Z. Bagiyarova F. Kussainova D. (2022). Prevalence of anxiety symptoms in women newly diagnosed with breast Cancer in Kazakhstan and its associated factors. Asian Pac. J. Cancer Prev. 23, 2813–2819. doi: 10.31557/APJCP.2022.23.8.2813, 36037138 PMC9741881

[ref44] Kumar R. Pasricha R. Gupta M. K. Ravi B. (2023). Psychosocial well-being and quality of life in women with breast cancer in foothills of North India. J. Cancer Res. Ther. 19, 1176–1185. doi: 10.4103/jcrt.jcrt_672_21, 37787281

[ref45] Lake P. W. Conley C. C. Pal T. Sutton S. K. Vadaparampil S. T. (2022). Anxiety and depression among black breast cancer survivors: examining the role of patient-provider communication and cultural values. Patient Educ. Couns. 105, 2391–2396. doi: 10.1016/j.pec.2021.12.020, 35440374 PMC10777429

[ref46] Lee M. Kim Y. H. Jeon M. J. (2015). Risk factors for negative impacts on sexual activity and function in younger breast cancer survivors. Psychooncology 24, 1097–1103. doi: 10.1002/pon.3772, 25690696

[ref47] Lee J. Lee M.-G. (2020). Effects of exercise interventions on breast cancer patients during adjuvant therapy: a systematic review and meta-analysis of randomized controlled trials. Cancer Nurs. 43, 115–125. doi: 10.1097/NCC.0000000000000682, 30601270

[ref48] Leo C. S. Y. Cheng L. J. Lam X. R. He H. (2024). Global prevalence and factors associated with preoperative depression in women undergoing breast surgery: a meta-analysis and meta-regression. Breast Cancer Res. Treat. 205, 425–438. doi: 10.1007/s10549-023-07216-3, 38492162

[ref49] Li J. Gao W. Yang Q. Cao F. (2021). Perceived stress, anxiety, and depression in treatment‐naïve women with breast cancer: a case‐control study. Psycho-Oncology 30, 231–239. doi: 10.1002/pon.5555, 32969126

[ref50] Li L. Yang Y. He J. Yi J. Wang Y. Zhang J. . (2015). Emotional suppression and depressive symptoms in women newly diagnosed with early breast cancer. BMC Womens Health 15:91. doi: 10.1186/s12905-015-0254-6, 26497200 PMC4620014

[ref51] Liu B. Wu X. Shi L. Li H. Wu D. Lai X. . (2021). Correlations of social isolation and anxiety and depression symptoms among patients with breast cancer of Heilongjiang province in China: the mediating role of social support. Nurs. Open 8, 1981–1989. doi: 10.1002/nop2.876, 33939294 PMC8186692

[ref52] Lopes C. Lopes-Conceição L. Fontes F. Ferreira A. Pereira S. Lunet N. . (2022). Prevalence and persistence of anxiety and depression over five years since breast cancer diagnosis—the NEON-BC prospective study. Curr. Oncol. (Toronto, Ont.) 29, 2141–2153. doi: 10.3390/curroncol29030173, 35323373 PMC8947204

[ref53] Mahlaq S. Lahlou L. Rammouz I. Abouqal R. Belayachi J. (2023). Factors associated with psychological burden of breast cancer in women in Morocco: cross-sectional study. BMC Womens Health 23:590. doi: 10.1186/s12905-023-02769-3, 37950247 PMC10638816

[ref54] Mao Y. Shi R. X. Gao L. M. Xu A. Y. Li J. N. Wang B. . (2025). Construction of a nomogram-based risk prediction model for depressive symptoms in middle-aged and young breast cancer patients. World J. Clin. Oncol. 16:102208. doi: 10.5306/wjco.v16.i4.102208, 40290700 PMC12019283

[ref56] Miller K. D. Nogueira L. Devasia T. Mariotto A. B. Yabroff K. R. Jemal A. . (2022). Cancer treatment and survivorship statistics, 2022. CA Cancer J. Clin. 72, 409–436. doi: 10.3322/caac.21731, 35736631

[ref57] Mitchell A. J. Vaze A. Rao S. (2009). Clinical diagnosis of depression in primary care: a meta-analysis. Lancet 374, 609–619. doi: 10.1016/s0140-6736(09)60879-5 19640579

[ref59] Msenga U. Ambikile J. S. Buluba S. E. (2025). Prevalence of anxiety and depression symptoms and associated factors among women diagnosed with breast cancer: a cross-sectional study at ocean road Cancer Institute in Dar es salaam-Tanzania. PLoS One 20:e0326749. doi: 10.1371/journal.pone.0326749, 40608778 PMC12225821

[ref60] Nho J. H. Kim S. R. Park M. H. Kweon S. S. (2018). Symptom clusters and quality of life in breast cancer survivors after cancer treatment in a tertiary hospital in Korea. Eur J Cancer Care (Engl) 27:e12919. doi: 10.1111/ecc.12919, 30253019

[ref61] Novakov I. Popovic-Petrovic S. Dugandžija T. Tatić M. (2019). Empirical validation of the integrative psychological group intervention for women with breast cancer–preliminary results. Vojnosanit. Pregl. 76, 1022–1028. doi: 10.2298/VSP171011004N

[ref62] Okati-Aliabad H. Ansari-Moghadam A. Mohammadi M. Kargar S. Shahraki-Sanavi F. (2022). The prevalence of anxiety and depression and its association with coping strategies, supportive care needs, and social support among women with breast cancer. Support Care Cancer 30, 703–710. doi: 10.1007/s00520-021-06477-2, 34365523

[ref63] Okubo R. Noguchi H. Hamazaki K. Sekiguchi M. Kinoshita T. Katsumata N. . (2018). Association between blood polyunsaturated fatty acid levels and depressive symptoms in breast cancer survivors. Prostaglandins Leukot. Essent. Fat. Acids 139, 9–13. doi: 10.1016/j.plefa.2018.11.002, 30471773

[ref64] Park E. M. Gelber S. Rosenberg S. M. Seah D. S. E. Schapira L. Come S. E. . (2018). Anxiety and depression in young women with metastatic breast cancer: a cross-sectional study. Psychosomatics 59, 251–258. doi: 10.1016/j.psym.2018.01.007, 29525523 PMC5935568

[ref65] Probst G. H. Gómez Penedo J. M. Flückiger C. (2025). Long-term efficacy of cognitive behavioural therapy for generalized anxiety disorder: a 2-8-year follow-up of two randomized controlled trials. Behav. Res. Ther. 191:104758. doi: 10.1016/j.brat.2025.104758, 40319554

[ref66] Puigpinós-Riera R. Graells-Sans A. Serral G. Continente X. Bargalló X. Domènech M. . (2018). Anxiety and depression in women with breast cancer: social and clinical determinants and influence of the social network and social support (DAMA cohort). Cancer Epidemiol. 55, 123–129. doi: 10.1016/j.canep.2018.06.002, 29940418

[ref67] Qureshi Z. Altaf F. Jamil A. Siddique R. (2025). Unlocking the mysteries of breast cancer: the role of epigenetics in diagnosis, treatment, and beyond. Am. J. Clin. Oncol. 48, 276–282. doi: 10.1097/COC.0000000000001177, 40025834

[ref68] Sadaqa D. Farraj A. Naseef H. Alsaid H. Al-Shami N. AbuKhalil A. D. (2022). Risk of developing depression among breast cancer patients in Palestine. BMC Cancer 22:295. doi: 10.1186/s12885-022-09420-8, 35313833 PMC8935763

[ref69] Schapira L. Zheng Y. Gelber S. I. Poorvu P. Ruddy K. J. Tamimi R. M. . (2022). Trajectories of fear of cancer recurrence in young breast cancer survivors. Cancer 128, 335–343. doi: 10.1002/cncr.33921, 34614212 PMC9397577

[ref70] Sharif Z. Harrison B. J. Davey C. G. Steward T. Schmaal L. Felmingham K. L. . (2026). Prevalence and predictors of anxiety and depression among breast cancer patients in Abbottabad, Pakistan. BMC Public Health 26:236. doi: 10.1186/s12889-025-25673-x

[ref71] Silverstein M. C. Lee C. N. Scherer L. D. Phommasathit C. Merrill A. L. Peters E. (2023). Operating on anxiety: negative affect toward breast Cancer and choosing contralateral prophylactic mastectomy. Med. Decis. Mak. 43, 152–163. doi: 10.1177/0272989X221121134, 36059240 PMC9898882

[ref72] So W. K. Marsh G. Ling W. M. Leung F. Y. Lo J. C. Yeung M. . (2010). Anxiety, depression and quality of life among Chinese breast cancer patients during adjuvant therapy. Eur. J. Oncol. Nurs. 14, 17–22. doi: 10.1016/j.ejon.2009.07.005, 19734087

[ref74] Tao F. Xu M. Zou Q. Tang L. Feng J. Li Z. (2023). Prevalence and severity of anxiety and depression in Chinese patients with breast cancer: a systematic review and meta-analysis. Front. Psych. 14:1080413. doi: 10.3389/fpsyt.2023.1080413, 37448492 PMC10336240

[ref75] Thakur M. Sharma R. Mishra A. K. Singh K. R. (2021). Prevalence and psychobiological correlates of depression among breast Cancer patients. Indian J. Surg. Oncol. 12, 251–257. doi: 10.1007/s13193-021-01296-7, 34295067 PMC8272766

[ref76] Tojal C. Costa R. (2015). Depressive symptoms and mental adjustment in women with breast cancer. Psychooncology 24, 1060–1065. doi: 10.1002/pon.3765, 25645194

[ref77] Tsaras K. Papathanasiou I. V. Mitsi D. Veneti A. Kelesi M. Zyga S. . (2018). Assessment of depression and anxiety in breast cancer patients: prevalence and associated factors. Asian Pac. J. Cancer Prev. 19, 1661–1669. doi: 10.22034/APJCP.2018.19.6.1661, 29938451 PMC6103579

[ref78] Turdaliyeva B. Karibayeva I. Zainal N. Z. Bagiyarova F. Kussainova D. (2022). Prevalence and associated factors of depression symptoms in women newly diagnosed with breast cancer in Kazakhstan. Asian Pac. J. Cancer Prev. 23, 2483–2489. doi: 10.31557/APJCP.2022.23.7.2483, 35901357 PMC9727346

[ref79] Valderrama Rios M. C. Sánchez Pedraza R. (2018). Anxiety and depression disorders in relation to the quality of life of breast cancer patients with locally advanced or disseminated stage. Rev Colomb Psiquiatr (Engl Ed) 47, 211–220. doi: 10.1016/j.rcp.2017.04.003, 30286843

[ref80] Wang R. He J. Cheng L. Huang Z. Miao X. Cheng X. . (2026). Association between composite dietary antioxidant index and depressive symptoms in breast cancer patients: the role of oxidative stress biomarkers in a cross-sectional study. Nutrients 18:2230. doi: 10.3390/nu18142230, 42514299 PMC13414930

[ref83] Willers N. Enzlin P. Neven P. Han S. N. (2025). Sexuality after breast cancer treatment: an experience of asymmetry. Womens Health (Lond) 21:17455057241310271. doi: 10.1177/17455057241310271, 40248903 PMC12035161

[ref84] Wondimagegnehu A. Abebe W. Abraha A. Teferra S. (2019). Depression and social support among breast cancer patients in Addis Ababa, Ethiopia. BMC Cancer 19:836. doi: 10.1186/s12885-019-6007-4, 31455282 PMC6712811

[ref86] Wulandari P. Livana P. H. (2022). Factors that influence the incidence of depression in breast cancer patients. Open Access Maced. J. Med. Sci. 10, 393–399. doi: 10.3889/oamjms.2022.7740

[ref87] Yan J. Wang F. Yu X. (2025). Effects of mindfulness therapy on breast cancer patients with depression or anxiety: a systematic review and meta-analysis. Actas Esp. Psiquiatr. 53, 422–432. doi: 10.62641/aep.v53i2.1949, 40071356 PMC11898256

[ref88] Zeilinger E. L. Oppenauer C. Knefel M. Kantor V. Schneckenreiter C. Lubowitzki S. . (2022). Prevalence of anxiety and depression in people with different types of cancer or haematologic malignancies: a cross-sectional study. Epidemiol. Psychiatr. Sci. 31:e74. doi: 10.1017/S2045796022000592, 36245424 PMC9583630

[ref89] Zhao R. Liu H. Gao J. (2022). Side effects of endocrine therapy are associated with depression and anxiety in breast Cancer patients accepting endocrine therapy: a cross-sectional study in China. Front. Psychol. 13:905459. doi: 10.3389/fpsyg.2022.905459, 35615194 PMC9125212

