# Supplementary material for: Prevalence of anxiety and depression in breast cancer patients: a meta-analysis
Source: Front Psychol. 2026 Oct 14;17:1869669. doi: 10.3389/fpsyg.2026.1869669 (PMC13713415; doi:10.3389/fpsyg.2026.1869669)
Supplement: Supplementary file 2 [file Supplementary_file_2.docx]

**Supplementary Material**

**Table S1** Search strategies.

| **Source** | **Strategy or screening approach** | **Records** |
| --- | --- | --- |
| PubMed | (("Breast Neoplasms"[Mesh] OR breast cancer[Title/Abstract] OR breast neoplasm*[Title/Abstract] OR breast tumo?r*[Title/Abstract] OR mammary cancer[Title/Abstract] OR mammary carcinoma[Title/Abstract])) AND (("Depression"[Mesh] OR "Anxiety"[Mesh] OR depression[Title/Abstract] OR depressive symptom*[Title/Abstract] OR depressive disorder*[Title/Abstract] OR anxiety[Title/Abstract] OR anxiety symptom*[Title/Abstract] OR anxiety disorder*[Title/Abstract] OR psychological distress[Title/Abstract])) AND ((prevalence[Title/Abstract] OR epidemiolog*[Title/Abstract] OR frequency[Title/Abstract] OR rate*[Title/Abstract] OR proportion*[Title/Abstract] OR burden[Title/Abstract] OR "cross-sectional"[Title/Abstract] OR "cross sectional"[Title/Abstract])) AND (("2015/01/01"[Date - Publication] : "2026/09/10"[Date - Publication])) | 2,156 |
| Web of Science | TS=(("breast cancer" OR "breast neoplasm*" OR "breast tumor*" OR "breast tumour*" OR "mammary cancer" OR "mammary carcinoma") AND (depression OR "depressive symptom*" OR "depressive disorder*" OR anxiety OR "anxiety symptom*" OR "anxiety disorder*" OR "psychological distress") AND (prevalence OR epidemiolog* OR frequency OR rate* OR proportion* OR burden OR "cross-sectional" OR "cross sectional")) AND PY=(2015-2026) | 4,027 |
| Embase | ('breast cancer'/exp OR 'breast cancer':ti,ab OR 'breast neoplasm*':ti,ab OR 'breast tumor*':ti,ab OR 'breast tumour*':ti,ab OR 'mammary cancer':ti,ab OR 'mammary carcinoma':ti,ab) AND ('depression'/exp OR 'anxiety'/exp OR depression:ti,ab OR 'depressive symptom*':ti,ab OR 'depressive disorder*':ti,ab OR anxiety:ti,ab OR 'anxiety symptom*':ti,ab OR 'anxiety disorder*':ti,ab OR 'psychological distress':ti,ab) AND (prevalence:ti,ab OR epidemiolog*:ti,ab OR frequency:ti,ab OR rate*:ti,ab OR proportion*:ti,ab OR burden:ti,ab OR 'cross-sectional':ti,ab OR 'cross sectional':ti,ab) AND [2015-2026]/py | 3,325 |
| Cochrane Library | #1 MeSH descriptor: [Breast Neoplasms] explode all trees #2 (breast cancer OR breast neoplasm* OR breast tumor* OR breast tumour* OR mammary cancer OR mammary carcinoma):ti,ab,kw #3 #1 OR #2 #4 MeSH descriptor: [Depression] explode all trees #5 MeSH descriptor: [Anxiety] explode all trees #6 (depression OR "depressive symptom*" OR "depressive disorder*" OR anxiety OR "anxiety symptom*" OR "anxiety disorder*" OR "psychological distress"):ti,ab,kw #7 #4 OR #5 OR #6 #8 (prevalence OR frequency OR rate* OR proportion* OR "cross-sectional" OR "cross sectional"):ti,ab,kw #9 #3 AND #7 AND #8 Publication Year from 2015 to 2025 | 1,485 |
| APA PsycINFO | ((TI("breast cancer" OR "breast neoplasm*" OR "breast tumor*" OR "breast tumour*" OR "mammary cancer" OR "mammary carcinoma") OR AB("breast cancer" OR "breast neoplasm*" OR "breast tumor*" OR "breast tumour*" OR "mammary cancer" OR "mammary carcinoma") OR KW("breast cancer" OR "breast neoplasm*" OR "breast tumor*" OR "breast tumour*" OR "mammary cancer" OR "mammary carcinoma")) AND (TI(depression OR "depressive symptom*" OR "depressive disorder*" OR anxiety OR "anxiety symptom*" OR "anxiety disorder*" OR "psychological distress") OR AB(depression OR "depressive symptom*" OR "depressive disorder*" OR anxiety OR "anxiety symptom*" OR "anxiety disorder*" OR "psychological distress") OR KW(depression OR "depressive symptom*" OR "depressive disorder*" OR anxiety OR "anxiety symptom*" OR "anxiety disorder*" OR "psychological distress")) AND (TI(prevalence OR epidemiolog* OR frequency OR rate* OR proportion* OR burden OR "cross-sectional" OR "cross sectional") OR AB(prevalence OR epidemiolog* OR frequency OR rate* OR proportion* OR burden OR "cross-sectional" OR "cross sectional") OR KW(prevalence OR epidemiolog* OR frequency OR rate* OR proportion* OR burden OR "cross-sectional" OR "cross sectional"))) Limiters: Publication Year 2015-2026 | 97 |
| Scopus | TITLE-ABS-KEY(("breast cancer" OR "breast neoplasm*" OR "breast tumor*" OR "breast tumour*" OR "mammary cancer" OR "mammary carcinoma") AND (depression OR "depressive symptom*" OR "depressive disorder*" OR anxiety OR "anxiety symptom*" OR "anxiety disorder*" OR "psychological distress") AND (prevalence OR epidemiolog* OR frequency OR rate* OR proportion* OR burden OR "cross-sectional" OR "cross sectional")) AND PUBYEAR > 2014 AND PUBYEAR < 2027 | 118 |

**Table S2** Subgroup analyses of anxiety and depression prevalence.

| **Subgroup** | **k** | **Prevalence** | **95% CI** | **I2 (%)** | **p within** | **k** | **Prevalence** | **95% CI** | **I2 (%)** | **p within** |
| --- | --- | --- | --- | --- | --- | --- | --- | --- | --- | --- |
| **Region** |  |  |  |  |  |  |  |  |  |  |
| Asia | 22 | 45.7% | 34.3%-57.4% | 98.4 | <0.001 | 29 | 49.4% | 41.0%-57.7% | 97.7 | <0.001 |
| Europe | 9 | 46.2% | 34.7%-57.9% | 98.4 | <0.001 | 12 | 33.2% | 25.8%-40.9% | 97.9 | <0.001 |
| Africa | 5 | 54.9% | 34.7%-74.3% | 98.0 | <0.001 | 4 | 59.1% | 52.1%-65.8% | 77.7 | 0.004 |
| North America | 2 | 40.4% | 35.7%-45.3% | 0.0 | 0.542 | 3 | 27.0% | 13.2%-43.6% | 94.0 | <0.001 |
| South America | 1 | 84.1% | 76.5%-90.5% | - | - | 1 | 25.2% | 17.4%-33.9% | - | - |
| **Between-region difference** |  |  |  |  | **p < 0.001** |  |  |  |  | **p < 0.001** |
| **Assessment tool** |  |  |  |  |  |  |  |  |  |  |
| BDI | - | - | - | - | - | 4 | 64.4% | 35.8%-88.4% | 97.9 | <0.001 |
| DASS-21 subscale | 4 | 54.8% | 46.8%-62.6% | 78.9 | 0.003 | 4 | 47.9% | 41.1%-54.7% | 71.5 | 0.015 |
| HADS subscale | 23 | 52.1% | 43.6%-60.4% | 98.2 | <0.001 | 25 | 39.8% | 30.7%-49.4% | 98.7 | <0.001 |
| Two-item screening scale | 2 | 39.2% | 25.8%-53.5% | 84.2 | 0.012 | 2 | 46.1% | 30.8%-61.8% | 86.9 | 0.006 |
| Other | 10 | 37.9% | 23.7%-53.2% | 97.9 | <0.001 | 14 | 45.6% | 36.1%-55.3% | 97.7 | <0.001 |
| **Between-tool difference** |  |  |  |  | **p = 0.102** |  |  |  |  | **p = 0.479** |
| **Study design** |  |  |  |  |  |  |  |  |  |  |
| Cross-sectional | 37 | 47.9% | 40.5%-55.4% | 98.3 | <0.001 | 45 | 44.3% | 37.5%-51.2% | 98.3 | <0.001 |
| Case-control | 1 | 38.3% | 29.8%-47.2% | - | - | 3 | 50.7% | 22.5%-78.6% | 98.9 | <0.001 |
| Cohort | 1 | 55.3% | 51.0%-59.6% | - | - | 1 | 25.5% | 21.8%-29.4% | - | - |
| **Between-design difference** |  |  |  |  | **p = 0.002** |  |  |  |  | **p < 0.001** |

Abbreviations: BDI, Beck Depression Inventory; CI, confidence interval; DASS-21, Depression Anxiety Stress Scales-21; HADS, Hospital Anxiety and Depression Scale; k, number of reports. Category-row p values test within-category heterogeneity; between-group p values are shown in the difference rows.

**Table S3** Study-level full-text exclusion and reconciliation log.

| **No.** | **Record ID** | **Report identifier** | **Assessment stage** | **Final/revision disposition** | **Reason category** | **Specific reason** |
| --- | --- | --- | --- | --- | --- | --- |
| 1 | LEG-001 | Hassan, M.R. | Full-text review | Excluded in the legacy full-text log | Association study without eligible prevalence | The report examined correlates of depression/anxiety rather than providing an eligible breast-cancer prevalence estimate. |
| 2 | LEG-002 | Jones, S.M.W. | Full-text review | Excluded in the legacy full-text log | No extractable prevalence | No depression or anxiety prevalence was reported; the report presented changes in scale scores before and after diagnosis. |
| 3 | LEG-003 | Kitano, A. | Full-text review | Excluded in the legacy full-text log | Screening/intervention question | The study evaluated the psychological effect of breast-cancer screening rather than prevalence in a breast-cancer cohort. |
| 4 | LEG-004 | Lou, Z. | Full-text review | Excluded in the legacy full-text log | Wrong population | The participants had benign breast masses rather than breast cancer. |
| 5 | LEG-005 | McSweeney, L.J. | Full-text review | Excluded in the legacy full-text log | Restricted treatment subgroup | Short report restricted to patients after radical surgery; no eligible general breast-cancer prevalence estimate was retained. |
| 6 | LEG-006 | Stafford, L. | Full-text review | Excluded in the legacy full-text log | Selected psychiatric-history subgroup | The sample was selected according to neuroticism or psychiatric history and did not represent the prespecified population. |
| 7 | LEG-007 | Alfonsson, S. | Full-text review | Excluded in the legacy full-text log | No single extractable time point | The same cohort was assessed at multiple time points and no single eligible prevalence estimate could be derived. |
| 8 | LEG-008 | Dutta, S.K. | Full-text review | Excluded in the legacy full-text log | Composite psychiatric outcome | The reported psychiatric prevalence combined depressive disorder with panic disorder and was not separable as depression or anxiety. |
| 9 | LEG-009 | Huang, C.Y. | Full-text review | Excluded in the legacy full-text log | Association study without eligible prevalence | Short report focused on resilience-related associations and did not provide an eligible prevalence estimate. |
| 10 | LEG-010 | Hubalek, M. | Full-text review | Excluded in the legacy full-text log | Association study without eligible prevalence | The report focused on associations with endocrine factors and functional well-being rather than an eligible prevalence estimate. |
| 11 | LEG-011 | Jacob, L. | Full-text review | Excluded in the legacy full-text log | Database/claims analysis | Secondary database analysis rather than a sampled observational prevalence survey. |
| 12 | LEG-012 | Lababidi, S. | Full-text review | Excluded in the legacy full-text log | Mixed cancer population | Breast cancer was pooled within a broader gynecological-cancer group and breast-cancer-specific prevalence was unavailable. |
| 13 | LEG-013 | Li, D.Z. | Full-text review | Excluded in the legacy full-text log | No extractable prevalence | Only questionnaire scores were reported; an event numerator and prevalence were unavailable. |
| 14 | LEG-014 | Mallika, S. | Full-text review | Excluded in the legacy full-text log | Mixed cancer population | Mixed cancer population without separately extractable breast-cancer prevalence. |
| 15 | LEG-015 | Marschner, N. | Full-text review | Excluded in the legacy full-text log | Restricted treatment subgroup | The estimate was restricted to a treatment-specific setting and did not meet the recorded eligibility definition. |
| 16 | LEG-016 | Palmer, S.C. | Full-text review | Excluded in the legacy full-text log | Selected clinical-need subgroup | Participants were selected for unmet physical or psychological needs; no eligible unselected prevalence estimate was retained. |
| 17 | LEG-017 | Palmer, S.C. | Full-text review | Excluded in the legacy full-text log | Selected clinical-need subgroup | A second report of an unmet-needs-selected sample; no eligible unselected prevalence estimate was retained. |
| 18 | LEG-018 | Ristevska-Dimitrovska, G. & Dejanova, B. | Full-text review | Excluded in the legacy full-text log | Unclear diagnostic category | Diagnostic categories included an adjustment-disorder depressive subtype and were not clearly separable; the legacy log also recorded no DOI. |
| 19 | LEG-019 | Vatvani, A.D. | Full-text review | Excluded in the legacy full-text log | No extractable prevalence | No depression/anxiety prevalence was reported; only the proportion classified as having no abnormality was presented. |
| 20 | LEG-020 | Srivastava, V. | Full-text review | Excluded in the legacy full-text log | No extractable prevalence | Only questionnaire scores were reported; an event numerator and prevalence were unavailable. |
| 21 | LEG-021 | Azegrar, M. | Full-text review | Excluded in the legacy full-text log | Restricted treatment subgroup | Population restricted to patients after radical surgery. |
| 22 | LEG-022 | Galindo-Vazquez, O. | Full-text review | Excluded in the legacy full-text log | Conference abstract | Conference abstract without sufficient full report data. |
| 23 | LEG-023 | Geyikci, R. | Full-text review | Excluded in the legacy full-text log | Association study without eligible prevalence | The study focused on demographic and socioeconomic correlates rather than providing an eligible prevalence estimate. |
| 24 | LEG-024 | Guermazi, F. | Full-text review | Excluded in the legacy full-text log | Association study without eligible prevalence | The study focused on economic, regional, and medication correlates rather than providing an eligible prevalence estimate. |
| 25 | LEG-025 | Lange, M. | Full-text review | Excluded in the legacy full-text log | Short report/non-prevalence focus | Short report focused primarily on cognitive status; no eligible prevalence estimate was retained. |
| 26 | LEG-026 | Maass, S.W.M.C. | Full-text review | Excluded in the legacy full-text log | No extractable prevalence | No numeric prevalence data were reported. |
| 27 | LEG-027 | Mertz, B.G. | Full-text review | Excluded in the legacy full-text log | Restricted disease-stage subgroup | Population restricted to ductal carcinoma in situ. |
| 28 | LEG-028 | Minowa, C | Full-text review | Excluded in the legacy full-text log | Mixed screening population/no prevalence | Mixed population of breast cancer, screening, and examination-seeking participants; only scale scores were reported. |
| 29 | LEG-029 | Park, E.M. | Full-text review | Re-evaluated and included in the primary meta-analysis | Re-evaluated and included | Originally excluded because the sample comprised young women with metastatic breast cancer; re-evaluated during revision and included as Park et al. (2018). |
| 30 | LEG-030 | Sharpe, N. | Full-text review | Excluded in the legacy full-text log | Association study without eligible prevalence | The report focused on correlates such as time since diagnosis and family/friend concerns rather than an eligible prevalence estimate. |
| 31 | LEG-031 | Silva, J. | Full-text review | Excluded in the legacy full-text log | Composite/unclear psychiatric outcome | The outcome combined several depressive or adjustment-related disorders and was not an eligible separable prevalence measure. |
| 32 | LEG-032 | Travado, L. | Full-text review | Excluded in the legacy full-text log | Conference abstract | Conference abstract without sufficient full report data. |
| 33 | LEG-033 | Cindy, C. | Full-text review | Excluded in the legacy full-text log | Association study without eligible prevalence | Cancer-pain association study reporting scale scores rather than an eligible prevalence estimate. |
| 34 | LEG-034 | Geske, S.J. | Full-text review | Excluded in the legacy full-text log | Restricted advanced-cancer population | Population restricted to advanced cancer and not separately documented as an eligible breast-cancer prevalence sample in the legacy log. |
| 35 | LEG-035 | Tancherla, A. | Full-text review | Excluded in the legacy full-text log | Short report/restricted treatment subgroup | Short report restricted to patients after mastectomy and adjuvant chemotherapy. |
| 36 | LEG-036 | Tiwari, A. | Full-text review | Excluded in the legacy full-text log | Survivor-only population | Population restricted to breast-cancer survivors under the recorded eligibility rule. |
| 37 | LEG-037 | Burgos, J. J. A. | Full-text review | Excluded in the legacy full-text log | Survivor-only population | Population restricted to breast-cancer survivors under the recorded eligibility rule. |
| 38 | LEG-038 | Grusdat, N. | Full-text review | Excluded in the legacy full-text log | Association study without eligible prevalence | The report examined pretreatment functional and mental-status correlates rather than an eligible prevalence estimate. |
| 39 | LEG-039 | Kyei, K.A. | Full-text review | Excluded in the legacy full-text log | Restricted treatment subgroup | Population restricted to patients receiving radiotherapy. |
| 40 | LEG-040 | Lan, B. | Full-text review | Excluded in the legacy full-text log | Postoperative-only population | Population restricted to postoperative breast-cancer patients. |
| 41 | LEG-041 | Shim, E. J. | Full-text review | Excluded in the legacy full-text log | Unclear severity/complex outcome | Depression severity categories were unclear and the outcome was influenced by multiple symptom domains. |
| 42 | LEG-042 | Basto, R. | Full-text review | Reconciled with an included report; retained as an audit entry | Re-evaluated and included | Originally logged as metastatic-only; reconciled with the final included R. Basto report and retained here as an audit trail rather than a final exclusion. |
| 43 | LEG-043 | Berrazaga, Y. | Full-text review | Excluded in the legacy full-text log | COVID-period/recovered mixed population | Sample mixed recovered participants and was restricted to the COVID-19 pandemic period. |
| 44 | LEG-044 | Kunhikkandy, A. A. | Full-text review | Excluded in the legacy full-text log | Short report | Short report without sufficient extractable data. |
| 45 | LEG-045 | Nakamura, Z. M. | Full-text review | Excluded in the legacy full-text log | Restricted treatment subgroup | Population restricted to patients receiving chemotherapy. |
| 46 | LEG-046 | Sghaier, S. | Full-text review | Excluded in the legacy full-text log | Restricted treatment subgroup | Part of the sample had undergone curative treatment; no eligible estimate was retained under the recorded rule. |
| 47 | LEG-047 | Singh, B.J. | Full-text review | Excluded in the legacy full-text log | Restricted advanced-treatment subgroup | Population restricted to advanced breast cancer receiving palliative treatment. |
| 48 | LEG-048 | Ando, M. | Full-text review | Excluded in the legacy full-text log | Outcomes not separable | Depression and anxiety were combined in the assessment tool and separate proportions could not be extracted. |
| 49 | LEG-049 | Ando, M.M. | Full-text review | Excluded in the legacy full-text log | Outcomes not separable | Second report with a combined depression/anxiety measure that did not permit separate proportions. |
| 50 | LEG-050 | Breidenbach, C. | Full-text review | Excluded in the legacy full-text log | Postoperative-only population | Population restricted to postoperative breast-cancer patients. |
| 51 | LEG-051 | Gul, S.K. | Full-text review | Advanced to quality appraisal; excluded at 16/20 | Advanced to quality appraisal | Originally logged as surgery-completed nonmetastatic patients; advanced to quality appraisal and excluded because the adapted score was 16/20. |
| 52 | LEG-052 | Krishnan, C | Full-text review | Excluded in the legacy full-text log | COVID-period population | Psychological health was assessed only during the COVID-19 pandemic period. |
| 53 | LEG-053 | Lan, B. | Full-text review | Excluded in the legacy full-text log | Restricted disease-stage subgroup | Population restricted to early-stage breast cancer. |
| 54 | LEG-054 | Lv, D. | Full-text review | Excluded in the legacy full-text log | Postoperative/restricted stage subgroup | Population restricted to postoperative early-stage breast cancer. |
| 55 | LEG-055 | Nakamura, Z. | Full-text review | Excluded in the legacy full-text log | Restricted treatment subgroup | Population restricted to patients after chemotherapy. |
| 56 | LEG-056 | Rodrigues, J. | Full-text review | Excluded in the legacy full-text log | Restricted disease-stage subgroup | Population restricted to early-stage breast cancer. |
| 57 | LEG-057 | Seneviratne, S. | Full-text review | Excluded in the legacy full-text log | Unclear severity category | Depression and anxiety severity categories were insufficiently defined. |
| 58 | LEG-058 | Yang, C.M. | Full-text review | Excluded in the legacy full-text log | Database/claims analysis | Insurance/claims database analysis rather than a sampled observational prevalence survey. |
| 59 | LEG-059 | Ertaş, H. | Full-text review | Excluded in the legacy full-text log | Restricted disease-stage subgroup | Population restricted to early-stage breast cancer. |
| 60 | LEG-060 | Karoui, M. | Full-text review | Excluded in the legacy full-text log | Association study without eligible prevalence | The report focused on sexual and marital correlates rather than an eligible prevalence estimate. |
| 61 | LEG-061 | Karveli, S. | Full-text review | Excluded in the legacy full-text log | Restricted disease-stage subgroup | Population restricted to nonmetastatic breast cancer under the recorded eligibility rule. |
| 62 | LEG-062 | Kooli, N. | Full-text review | Excluded in the legacy full-text log | Mixed cancer population | Gynecological-cancer population without separately extractable breast-cancer prevalence. |
| 63 | LEG-063 | Prasanthi, J. | Full-text review | Excluded in the legacy full-text log | Association study without eligible prevalence | The report focused on somatic symptoms, fear, and other correlates rather than an eligible prevalence estimate. |
| 64 | LEG-064 | Zaouali, F. | Full-text review | Excluded in the legacy full-text log | Short report | Short report without sufficient extractable data. |
| 65 | LEG-065 | Freeman, J. | Full-text review | Excluded in the legacy full-text log | No extractable prevalence | No directly extractable prevalence estimate was reported. |
| 66 | LEG-066 | Fteropoulli, T. | Full-text review | Excluded in the legacy full-text log | Postoperative-only population | Population restricted to postoperative breast-cancer patients. |
| 67 | LEG-067 | Jayarajah, U. | Full-text review | Excluded in the legacy full-text log | Short report | Short report without sufficient extractable data. |
| 68 | LEG-068 | Manchiraju, H. | Full-text review | Excluded in the legacy full-text log | Short report | Short report without sufficient extractable data. |
| 69 | LEG-069 | Oliveira, M.D. | Full-text review | Excluded in the legacy full-text log | Postoperative-only population | Population restricted to postoperative breast-cancer patients. |
| 70 | LEG-070 | Ruddy, K. | Full-text review | Excluded in the legacy full-text log | Short report | Short report without sufficient extractable data. |
| 71 | LEG-071 | Syarif, H. | Full-text review | Excluded in the legacy full-text log | Restricted treatment subgroup | Population restricted to patients receiving chemotherapy. |
| 72 | LEG-072 | Stearns, V. | Full-text review | Excluded in the legacy full-text log | Restricted disease-stage subgroup | Population restricted to metastatic breast cancer under the recorded eligibility rule. |
| 73 | ORG-Q-001 | A. Wondimagegnehu | Quality appraisal | Excluded after quality appraisal | Quality threshold not met | Adapted 10-domain JBI-informed rubric score 17/20; prespecified inclusion threshold >17. Domains below full score: II=0, III=1. |
| 74 | ORG-Q-004 | S. K. Gul, | Quality appraisal | Excluded after quality appraisal | Quality threshold not met | Adapted 10-domain JBI-informed rubric score 16/20; prespecified inclusion threshold >17. Domains below full score: II=0, III=0. |
| 75 | ORG-Q-013 | M. K. Jang | Quality appraisal | Excluded after quality appraisal | Quality threshold not met | Adapted 10-domain JBI-informed rubric score 17/20; prespecified inclusion threshold >17. Domains below full score: II=0, III=1. |
| 76 | ORG-Q-016 | M. H. Antoni | Quality appraisal | Excluded after quality appraisal | Quality threshold not met | Adapted 10-domain JBI-informed rubric score 17/20; prespecified inclusion threshold >17. Domains below full score: II=0, III=1. |
| 77 | ORG-Q-021 | Nuworza Kugbey | Quality appraisal | Excluded after quality appraisal | Quality threshold not met | Adapted 10-domain JBI-informed rubric score 17/20; prespecified inclusion threshold >17. Domains below full score: II=1, III=1, IV=1. |
| 78 | ORG-Q-024 | E. Podvorica | Quality appraisal | Excluded after quality appraisal | Quality threshold not met | Adapted 10-domain JBI-informed rubric score 17/20; prespecified inclusion threshold >17. Domains below full score: II=0, VII=1. |
| 79 | ORG-Q-033 | S. Y. Jeon | Quality appraisal | Excluded after quality appraisal | Quality threshold not met | Adapted 10-domain JBI-informed rubric score 17/20; prespecified inclusion threshold >17. Domains below full score: II=0, III=1. |
| 80 | ORG-Q-040 | S. H. Al-Ghabeesh | Quality appraisal | Excluded after quality appraisal | Quality threshold not met | Adapted 10-domain JBI-informed rubric score 17/20; prespecified inclusion threshold >17. Domains below full score: II=0, X=1. |
| 81 | ORG-Q-042 | H. H. Faye-Schjøll | Quality appraisal | Excluded after quality appraisal | Quality threshold not met | Adapted 10-domain JBI-informed rubric score 16/20; prespecified inclusion threshold >17. Domains below full score: II=0, III=0. |
| 82 | ORG-Q-044 | Y. T. Cheung | Quality appraisal | Excluded after quality appraisal | Quality threshold not met | Adapted 10-domain JBI-informed rubric score 17/20; prespecified inclusion threshold >17. Domains below full score: II=0, IX=1. |
| 83 | ORG-Q-045 | J. Cvetkovic | Quality appraisal | Excluded after quality appraisal | Quality threshold not met | Adapted 10-domain JBI-informed rubric score 15/20; prespecified inclusion threshold >17. Domains below full score: II=0, III=1, VIII=0. |
| 84 | ORG-Q-047 | M. Gold | Quality appraisal | Excluded after quality appraisal | Quality threshold not met | Adapted 10-domain JBI-informed rubric score 17/20; prespecified inclusion threshold >17. Domains below full score: II=0, III=1. |
| 85 | ORG-N-004 | D. M. Howell | Quality appraisal | Excluded after quality appraisal | Quality threshold not met | Newcastle-Ottawa Scale score 5/9; prespecified inclusion threshold >7. Study-level NOS total retained from the original appraisal file. |
| 86 | ORG-N-005 | H. Naik | Quality appraisal | Excluded after quality appraisal | Quality threshold not met | Newcastle-Ottawa Scale score 6/9; prespecified inclusion threshold >7. Study-level NOS total retained from the original appraisal file. |
| 87 | REV-Q-011 | L. A. Hafiz | Quality appraisal | Excluded after quality appraisal | Quality threshold not met | Adapted 10-domain JBI-informed rubric score 17/20; prespecified inclusion threshold >17. online survey; eligibility and representativeness limited |
| 88 | REV-N-002 | F. Wang | Quality appraisal | Excluded after quality appraisal | Quality threshold not met | Newcastle-Ottawa Scale score 7/9; prespecified inclusion threshold >7. The main analysis used only the baseline before radiotherapy. |
| 89 | REV-N-003 | M. Konieczny | Quality appraisal | Excluded after quality appraisal | Quality threshold not met | Newcastle-Ottawa Scale score 7/9; prespecified inclusion threshold >7. The primary analysis used only the baseline data before chemotherapy. |

This table contains 89 audit entries: 72 decisions retained in the contemporaneous legacy full-text log and 17 exclusions after quality appraisal. The legacy source retained first-author identifiers and reasons but not complete title/year/DOI fields for every record; identifiers are therefore reproduced as recorded and missing bibliographic fields were not reconstructed. Park was re-evaluated and included, Basto was reconciled with an included report while the legacy entry was retained, and Gul advanced to quality appraisal but was excluded at 16/20.

**Table S4** Quality-appraisal methodology and report-level decisions. **(a).** Scoring rules; **(b).** Report-level scores and decisions.

**(a)**

| **Framework** | **Domain/item** | **0 points** | **1 point** | **2 points** |
| --- | --- | --- | --- | --- |
| Adapted 10-domain JBI-informed rubric | I Clear objective and rationale | Absent, not reported, or major concern | Partially reported or partly adequate | Clearly reported and adequate |
| Adapted 10-domain JBI-informed rubric | II Sampling frame and sampling method | Absent, not reported, or major concern | Partially reported or partly adequate | Clearly reported and adequate |
| Adapted 10-domain JBI-informed rubric | III Inclusion and exclusion criteria | Absent, not reported, or major concern | Partially reported or partly adequate | Clearly reported and adequate |
| Adapted 10-domain JBI-informed rubric | IV Description of sample characteristics | Absent, not reported, or major concern | Partially reported or partly adequate | Clearly reported and adequate |
| Adapted 10-domain JBI-informed rubric | V Valid and reliable measurement instrument | Absent, not reported, or major concern | Partially reported or partly adequate | Clearly reported and adequate |
| Adapted 10-domain JBI-informed rubric | VI Standardized or verified data collection | Absent, not reported, or major concern | Partially reported or partly adequate | Clearly reported and adequate |
| Adapted 10-domain JBI-informed rubric | VII Ethical approval | Absent, not reported, or major concern | Partially reported or partly adequate | Clearly reported and adequate |
| Adapted 10-domain JBI-informed rubric | VIII Statistical methods | Absent, not reported, or major concern | Partially reported or partly adequate | Clearly reported and adequate |
| Adapted 10-domain JBI-informed rubric | IX Results analysis and separation of inference | Absent, not reported, or major concern | Partially reported or partly adequate | Clearly reported and adequate |
| Adapted 10-domain JBI-informed rubric | X Interpretation and study value | Absent, not reported, or major concern | Partially reported or partly adequate | Clearly reported and adequate |
| Cross-sectional total | 10 domains × 0-2 points | Range 0-20 | Primary-analysis inclusion: >17 | A score <18 means 0-17 and was excluded |
| Important terminology | This is an adapted JBI-informed quantitative rubric | It is not the unmodified standard JBI checklist | Item-level scores are reported for transparency | No scores were retrospectively changed |
| Case-control/cohort | Newcastle-Ottawa Scale | Range 0-9 | Primary-analysis inclusion: >7 | Scores 0-7 were excluded |
| Decision rule | Thresholds were applied before the final synthesis | 52 of 69 quality-assessed reports were included | 17 were excluded after quality appraisal | See Supplementary Table S4 |

**(b)**

| **Record ID** | **First author** | **Design** | **Appraisal framework** | **Score** | **Maximum** | **Inclusion threshold** | **Final decision** | **Item-level note** |
| --- | --- | --- | --- | --- | --- | --- | --- | --- |
| ORG-Q-001 | A. Wondimagegnehu | Cross-sectional | Adapted 10-domain JBI-informed rubric | 17 | 20 | >17 | Excluded after quality appraisal | Domains below full score: II=0, III=1. |
| ORG-Q-002 | R. Okubo, H. Noguchi | Cross-sectional | Adapted 10-domain JBI-informed rubric | 18 | 20 | >17 | Included | Domains below full score: II=0. |
| ORG-Q-003 | A. Al-Fahdi | Cross-sectional | Adapted 10-domain JBI-informed rubric | 19 | 20 | >17 | Included | Domains below full score: II=1. |
| ORG-Q-004 | S. K. Gul, | Cross-sectional | Adapted 10-domain JBI-informed rubric | 16 | 20 | >17 | Excluded after quality appraisal | Domains below full score: II=0, III=0. |
| ORG-Q-005 | Y. Q. Guo | Cross-sectional | Adapted 10-domain JBI-informed rubric | 20 | 20 | >17 | Included | All ten domains received full credit. |
| ORG-Q-006 | R. Kumar | Cross-sectional | Adapted 10-domain JBI-informed rubric | 19 | 20 | >17 | Included | Domains below full score: II=1. |
| ORG-Q-007 | S. Mahalq | Cross-sectional | Adapted 10-domain JBI-informed rubric | 18 | 20 | >17 | Included | Domains below full score: II=0. |
| ORG-Q-008 | M. Buyuksimsek | Cross-sectional | Adapted 10-domain JBI-informed rubric | 18 | 20 | >17 | Included | Domains below full score: II=0. |
| ORG-Q-009 | H. Joulaei | Cross-sectional | Adapted 10-domain JBI-informed rubric | 19 | 20 | >17 | Included | Domains below full score: II=1. |
| ORG-Q-010 | K. Al Alawi | Cross-sectional | Adapted 10-domain JBI-informed rubric | 19 | 20 | >17 | Included | Domains below full score: II=1. |
| ORG-Q-011 | R. Puigpinós-Riera | Cross-sectional | Adapted 10-domain JBI-informed rubric | 19 | 20 | >17 | Included | Domains below full score: II=1. |
| ORG-Q-012 | N. Elamin | Cross-sectional | Adapted 10-domain JBI-informed rubric | 19 | 20 | >17 | Included | Domains below full score: II=1. |
| ORG-Q-013 | M. K. Jang | Cross-sectional | Adapted 10-domain JBI-informed rubric | 17 | 20 | >17 | Excluded after quality appraisal | Domains below full score: II=0, III=1. |
| ORG-Q-014 | R. Botto | Cross-sectional | Adapted 10-domain JBI-informed rubric | 18 | 20 | >17 | Included | Domains below full score: II=0. |
| ORG-Q-015 | R. Akel | Cross-sectional | Adapted 10-domain JBI-informed rubric | 18 | 20 | >17 | Included | Domains below full score: II=0. |
| ORG-Q-016 | M. H. Antoni | Cross-sectional | Adapted 10-domain JBI-informed rubric | 17 | 20 | >17 | Excluded after quality appraisal | Domains below full score: II=0, III=1. |
| ORG-Q-017 | E. C. Fradelos | Cross-sectional | Adapted 10-domain JBI-informed rubric | 19 | 20 | >17 | Included | Domains below full score: III=1. |
| ORG-Q-018 | H. B. Ju | Cross-sectional | Adapted 10-domain JBI-informed rubric | 19 | 20 | >17 | Included | Domains below full score: II=1. |
| ORG-Q-019 | J. H. Nho | Cross-sectional | Adapted 10-domain JBI-informed rubric | 18 | 20 | >17 | Included | Domains below full score: II=0. |
| ORG-Q-020 | Nashid Islam | Cross-sectional | Adapted 10-domain JBI-informed rubric | 18 | 20 | >17 | Included | Domains below full score: II=0. |
| ORG-Q-021 | Nuworza Kugbey | Cross-sectional | Adapted 10-domain JBI-informed rubric | 17 | 20 | >17 | Excluded after quality appraisal | Domains below full score: II=1, III=1, IV=1. |
| ORG-Q-022 | Paige W. Lakea, | Cross-sectional | Adapted 10-domain JBI-informed rubric | 18 | 20 | >17 | Included | Domains below full score: II=0. |
| ORG-Q-023 | H. Okati-Aliabad, | Cross-sectional | Adapted 10-domain JBI-informed rubric | 18 | 20 | >17 | Included | Domains below full score: II=1, III=1. |
| ORG-Q-024 | E. Podvorica | Cross-sectional | Adapted 10-domain JBI-informed rubric | 17 | 20 | >17 | Excluded after quality appraisal | Domains below full score: II=0, VII=1. |
| ORG-Q-025 | D. Sadaqa | Cross-sectional | Adapted 10-domain JBI-informed rubric | 18 | 20 | >17 | Included | Domains below full score: II=0. |
| ORG-Q-026 | P. Wulandari | Cross-sectional | Adapted 10-domain JBI-informed rubric | 18 | 20 | >17 | Included | Domains below full score: II=1, III=1. |
| ORG-Q-027 | E. L. Zeilinger | Cross-sectional | Adapted 10-domain JBI-informed rubric | 18 | 20 | >17 | Included | Domains below full score: II=0. |
| ORG-Q-028 | R. Zhao | Cross-sectional | Adapted 10-domain JBI-informed rubric | 18 | 20 | >17 | Included | Domains below full score: II=0. |
| ORG-Q-029 | K. Hajian-Tilaki | Cross-sectional | Adapted 10-domain JBI-informed rubric | 18 | 20 | >17 | Included | Domains below full score: II=0. |
| ORG-Q-030 | P. Aggeli | Cross-sectional | Adapted 10-domain JBI-informed rubric | 18 | 20 | >17 | Included | Domains below full score: II=0. |
| ORG-Q-031 | S. V. Doubova | Cross-sectional | Adapted 10-domain JBI-informed rubric | 18 | 20 | >17 | Included | Domains below full score: II=0. |
| ORG-Q-032 | A. Hajj | Cross-sectional | Adapted 10-domain JBI-informed rubric | 19 | 20 | >17 | Included | Domains below full score: II=1. |
| ORG-Q-033 | S. Y. Jeon | Cross-sectional | Adapted 10-domain JBI-informed rubric | 17 | 20 | >17 | Excluded after quality appraisal | Domains below full score: II=0, III=1. |
| ORG-Q-034 | B. Liu | Cross-sectional | Adapted 10-domain JBI-informed rubric | 18 | 20 | >17 | Included | Domains below full score: II=1, III=1. |
| ORG-Q-035 | M. Abu-Helalah | Cross-sectional | Adapted 10-domain JBI-informed rubric | 19 | 20 | >17 | Included | Domains below full score: II=1. |
| ORG-Q-036 | R. Basto | Cross-sectional | Adapted 10-domain JBI-informed rubric | 19 | 20 | >17 | Included | Domains below full score: III=1. |
| ORG-Q-037 | W. Belay | Cross-sectional | Adapted 10-domain JBI-informed rubric | 18 | 20 | >17 | Included | Domains below full score: II=0. |
| ORG-Q-038 | M. C. Cáceres | Cross-sectional | Adapted 10-domain JBI-informed rubric | 18 | 20 | >17 | Included | Domains below full score: II=0. |
| ORG-Q-039 | K. Tsaras | Cross-sectional | Adapted 10-domain JBI-informed rubric | 18 | 20 | >17 | Included | Domains below full score: II=1, III=1. |
| ORG-Q-040 | S. H. Al-Ghabeesh | Cross-sectional | Adapted 10-domain JBI-informed rubric | 17 | 20 | >17 | Excluded after quality appraisal | Domains below full score: II=0, X=1. |
| ORG-Q-041 | M. C. Valderrama Rios | Cross-sectional | Adapted 10-domain JBI-informed rubric | 19 | 20 | >17 | Included | Domains below full score: II=1. |
| ORG-Q-042 | H. H. Faye-Schjøll | Cross-sectional | Adapted 10-domain JBI-informed rubric | 16 | 20 | >17 | Excluded after quality appraisal | Domains below full score: II=0, III=0. |
| ORG-Q-043 | T. Akechi | Cross-sectional | Adapted 10-domain JBI-informed rubric | 20 | 20 | >17 | Included | All ten domains received full credit. |
| ORG-Q-044 | Y. T. Cheung | Cross-sectional | Adapted 10-domain JBI-informed rubric | 17 | 20 | >17 | Excluded after quality appraisal | Domains below full score: II=0, IX=1. |
| ORG-Q-045 | J. Cvetkovic | Cross-sectional | Adapted 10-domain JBI-informed rubric | 15 | 20 | >17 | Excluded after quality appraisal | Domains below full score: II=0, III=1, VIII=0. |
| ORG-Q-046 | O. A. Fatiregun | Cross-sectional | Adapted 10-domain JBI-informed rubric | 19 | 20 | >17 | Included | Domains below full score: IV=1. |
| ORG-Q-047 | M. Gold | Cross-sectional | Adapted 10-domain JBI-informed rubric | 17 | 20 | >17 | Excluded after quality appraisal | Domains below full score: II=0, III=1. |
| ORG-Q-048 | C. Tojal | Cross-sectional | Adapted 10-domain JBI-informed rubric | 18 | 20 | >17 | Included | Domains below full score: II=1, III=1. |
| ORG-N-001 | L. Li | Case-control | Newcastle-Ottawa Scale | 8 | 9 | >7 | Included | Study-level NOS total retained from the original appraisal file. |
| ORG-N-002 | D. Doege | Case-control | Newcastle-Ottawa Scale | 8 | 9 | >7 | Included | Study-level NOS total retained from the original appraisal file. |
| ORG-N-003 | C. Lopes | Cohort | Newcastle-Ottawa Scale | 8 | 9 | >7 | Included | Study-level NOS total retained from the original appraisal file. |
| ORG-N-004 | D. M. Howell | Cohort | Newcastle-Ottawa Scale | 5 | 9 | >7 | Excluded after quality appraisal | Study-level NOS total retained from the original appraisal file. |
| ORG-N-005 | H. Naik | Cohort | Newcastle-Ottawa Scale | 6 | 9 | >7 | Excluded after quality appraisal | Study-level NOS total retained from the original appraisal file. |
| REV-Q-001 | C. Civilotti | Cross-sectional | Adapted 10-domain JBI-informed rubric | 19 | 20 | >17 | Included | consecutive diagnostic sample; no random sampling |
| REV-Q-002 | E. M. Park | Cross-sectional | Adapted 10-domain JBI-informed rubric | 18 | 20 | >17 | Included | special young de novo metastatic sample; sampling method not random |
| REV-Q-003 | H. A. Alagizy | Cross-sectional | Adapted 10-domain JBI-informed rubric | 18 | 20 | >17 | Included | hospital sample; random sampling not stated |
| REV-Q-004 | I. Karibayeva | Cross-sectional | Adapted 10-domain JBI-informed rubric | 18 | 20 | >17 | Included | same cohort as Turdaliyeva; outcome-specific report |
| REV-Q-005 | B. Turdaliyeva | Cross-sectional | Adapted 10-domain JBI-informed rubric | 18 | 20 | >17 | Included | same cohort as Karibayeva; outcome-specific report |
| REV-Q-006 | Y. Mao | Cross-sectional | Adapted 10-domain JBI-informed rubric | 18 | 20 | >17 | Included | two tertiary hospitals; non-random recruitment |
| REV-Q-007 | U. Msenga | Cross-sectional | Adapted 10-domain JBI-informed rubric | 19 | 20 | >17 | Included | consecutive sampling at Ocean Road Cancer Institute |
| REV-Q-008 | B. Hessami | Cross-sectional | Adapted 10-domain JBI-informed rubric | 19 | 20 | >17 | Included | selection process described |
| REV-Q-009 | Z. Sharif | Cross-sectional | Adapted 10-domain JBI-informed rubric | 18 | 20 | >17 | Included | two-centre clinical sample; random sampling not stated |
| REV-Q-010 | İ. Gökmen | Cross-sectional | Adapted 10-domain JBI-informed rubric | 19 | 20 | >17 | Included | multicenter structured recruitment and medical-record verification |
| REV-Q-011 | L. A. Hafiz | Cross-sectional | Adapted 10-domain JBI-informed rubric | 17 | 20 | >17 | Excluded after quality appraisal | online survey; eligibility and representativeness limited |
| REV-Q-012 | S. M. Al Harrasi | Cross-sectional | Adapted 10-domain JBI-informed rubric | 19 | 20 | >17 | Included | survivor population and recruitment described |
| REV-Q-013 | R. Wang | Cross-sectional | Adapted 10-domain JBI-informed rubric | 18 | 20 | >17 | Included | cross-sectional clinical sample; random sampling not stated |
| REV-N-001 | J. Li | Case-control | Newcastle-Ottawa Scale | 8 | 9 | >7 | Included | breast-cancer case group n=120 |
| REV-N-002 | F. Wang | Cohort | Newcastle-Ottawa Scale | 7 | 9 | >7 | Excluded after quality appraisal | The main analysis only used the baseline data before radiotherapy |
| REV-N-003 | M. Konieczny | Cohort | Newcastle-Ottawa Scale | 7 | 9 | >7 | Excluded after quality appraisal | The primary analysis used only the baseline data before chemotherapy |

**Table S5** Quality threshold sensitivity analysis.

| **Outcome** | **Analysis** | **Reports** | **Participants** | **Pooled prevalence** | **95% CI** | **I2** | **tau2** |
| --- | --- | --- | --- | --- | --- | --- | --- |
| Depression | Primary analysis | 49 | 16,504 | 44.3% | 38.3%-50.4% | 98.3% | 0.0456 |
| Depression | All quality-appraised reports | 66 | 31,462 | 44.6% | 39.6%-49.6% | 98.6% | 0.0416 |
| Anxiety | Primary analysis | 39 | 11,536 | 47.9% | 40.9%-54.9% | 98.2% | 0.0490 |
| Anxiety | All quality-appraised reports | 53 | 25,751 | 48.1% | 41.0%-55.3% | 99.1% | 0.0699 |

Sensitivity analyses re-included all otherwise eligible quality-appraised reports with extractable event counts and denominators. The primary estimates were materially unchanged.

Note: All 69 quality-appraised reports were considered. Reports contributed only to outcomes with an eligible numerator and denominator. For longitudinal reports, only pretreatment or baseline observations were used. Random-effects estimates used the Freeman-Tukey double-arcsine transformation and DerSimonian-Laird model. Absolute changes were 0.27 percentage points for depression and 0.24 percentage points for anxiety.

**Figure S1** Mild depression.**(a).** Forest plot; **(b).** Funnel plot**; (c).** Leave-one-out analysis.

**Figure S2** Moderate depression. **(a).** Forest plot; **(b).** Funnel plot; **(c).** Leave-one-out analysis

**Figure S3** Severe depression. **(a).** Forest plot; **(b).** Funnel plot; **(c).** Leave-one-out analysis.

**Figure S4** Mild anxiety. **(a).**Forest plot; **(b).** Funnel plot; **(c).** leave-one-out analysis.

**Figure S5 Moderate anxiety. (a).**Forest plot; **(b).** Funnel plot; **(c).** leave-one-out analysis.

**Figure S6** Severe anxiety. **(a).**Forest plot; **(b).** Funnel plot; **(c).** leave-one-out analysis.

**Figure S7** Study-level screening and quality-appraisal dispositions.

Categories summarize the retained legacy full-text log and the final quality-appraisal exclusions. Re-evaluated and reconciled records are displayed separately to preserve the audit trail.

**Figure S8** Quality-appraisal score distributions and final decisions.

Panel **(a)** shows the adapted 10-domain JBI-informed rubric for cross-sectional reports; panel **(b)** shows Newcastle–Ottawa Scale scores for case-control/cohort reports. Primary-analysis thresholds were >17/20 and >7/9, respectively.

**Figure S9** Quality-threshold sensitivity analysis including all quality-appraised reports: depression.

**Figure S10** Quality-threshold sensitivity analysis including all quality-appraised reports: anxiety.

**Figure S11** Comparison of primary and all-quality-appraised pooled estimates.
